# Investigation of α,ω-Disubstituted Polyamine-Cholic Acid Conjugates Identifies Hyodeoxycholic and Chenodeoxycholic Scaffolds as Non-Toxic, Potent Antimicrobials

**DOI:** 10.3390/antibiotics12020404

**Published:** 2023-02-17

**Authors:** Kenneth Sue, Melissa M. Cadelis, Thomas Troia, Florent Rouvier, Marie-Lise Bourguet-Kondracki, Jean Michel Brunel, Brent R. Copp

**Affiliations:** 1 School of Chemical Sciences, The University of Auckland, Private Bag 92019, Auckland 1142, New Zealand; 2 UMR MD1 “Membranes et Cibles Therapeutiques”, U1261 INSERM, Faculté de Pharmacie, Aix-Marseille Universite, 27 bd Jean Moulin, 13385 Marseille, France; 3 Laboratoire Molécules de Communication et Adaptation des Micro-Organismes, UMR 7245 CNRS, Muséum National d’Histoire Naturelle, 57 Rue Cuvier (C.P. 54), 75005 Paris, France

**Keywords:** bile acids, hyodeoxycholic acid, polyamine, antimicrobial, MRSA, antifungal

## Abstract

With the increased incidence of antibiotic resistance, the discovery and development of new antibacterials is of increasing importance and urgency. The report of the natural product antibiotic squalamine in 1993 has stimulated a lot of interest in the study of structurally simplified cholic acid-polyamine derivatives. We report the synthesis of a focused set of deoxycholic acid-polyamine conjugates and the identification of hyodeoxycholic acid derivatives as being potently active towards *S. aureus* MRSA and some fungal strains, but with no attendant cytotoxicity or hemolytic properties. Analogue **7e** exhibited bactericidal activity towards a range of Gram-positive bacteria, while preliminary investigation of its mechanism of action ruled out the bacterial membrane as being a primary cellular target as determined using an ATP-release bioluminescence assay.

## 1. Introduction

**Figure 1:**
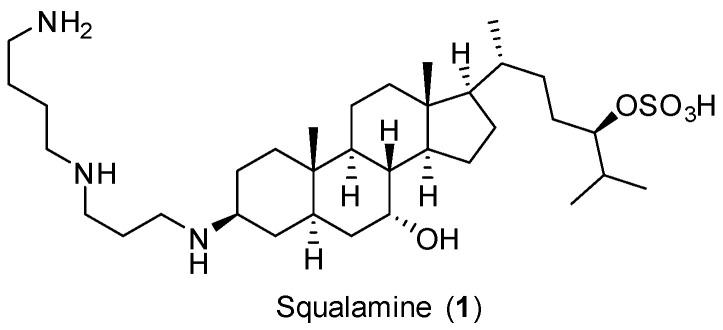
The structure of squalamine (**1**).

Natural products have a proven track record of being an excellent source of novel antibiotics or molecules that provide inspiration for the development of new therapeutics [1,2]. Squalamine (**1**) is an example of a natural product antibacterial drug lead, originally isolated from tissues of the dogfish shark *Squalus acanthias* (Figure 1) [3,4]. The unusual aminosterol exhibits broad-spectrum activity towards Gram-positive and Gram-negative bacteria as well as fungi and protozoa. The presence of both water-soluble groups (spermine and sulfate) and a lipophilic sterol core suggest that squalamine acts as a cationic amphiphilic antimicrobial targeting bacterial membranes, disruption of which leads to bacteria cell death [5].

Closer examination of the interaction of squalamine with eukaryotic and prokaryotic membranes identified the natural product to be more selective for binding to the latter and that interaction with LPS-containing membranes was calcium ion dependent [5,6]. Squalamine induced a rapid release of intracellular ATP from Gram-positive bacteria and led to disruption of the bacterial membrane as observed in TEM images [5,7]. Weak hemolytic activities have been reported for squalamine, with an EC_50_ of 80 μM for hemoglobin release from erythrocytes [8] and an EC_50_ 51 μM for propidium iodide entry into B lymphoma Wehi-231 cells [6], identifying it as a selective antibiotic lead for future development. In addition to demonstrating intrinsic antimicrobial properties, squalamine is also able to enhance the action of legacy antibiotics against Gram-negative bacteria [9]. In addition to membrane disrupting properties, a recent report has identified squalamine and structurally related mimics as inhibitors of the glycosyltransferase activity of *Escherichia coli* penicillin-binding protein PBP1b [10].

**Figure 2:**
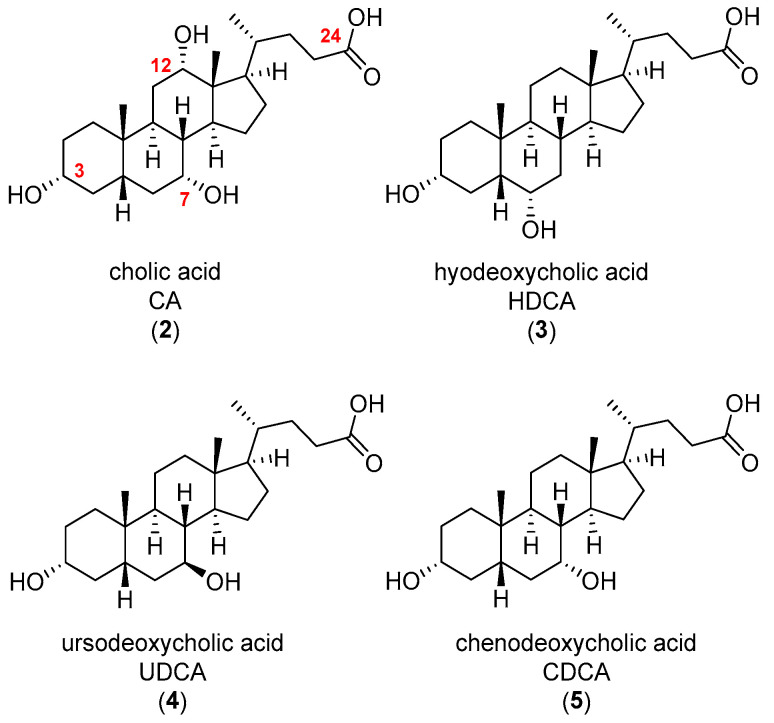
Structures of cholic (**2**), hyodeoxycholic (**3**), ursodeoxycholic (**4**) and chenodeoxycholic (**5**) acids.

The initial report of the structure and biological activities of squalamine has stimulated widespread interest in the discovery of mimics that are structurally simplified and more easily prepared, as recently reviewed [11,12]. Many studies of squalamine mimics have used bile acids e.g., cholic acid (CA) (**2**), as the steroidal scaffold, due to their plentiful supply and the availability of a number of related structures e.g., (7)-deoxycholic acid, and isomers hyodeoxycholic acid (HDCA) (**3**), ursodeoxycholic acid (UDCA) (**4**) and chenodeoxycholic acid (CDCA) (**5**) (Figure 2).

The particular position and/or stereochemistry of hydroxyl substitution on the cholic acid imparts ‘facial amphiphilicity’ [13,14,15,16], an attribute that has been exploited to develop bile acid-amine conjugates that exhibit wide ranging biological activities including acting as synthetic ionophores [17,18], plasmid transfection reagents [16,19], and antimicrobials [20,21,22,23,24]. In the case of the latter activity, cholic acid-amine conjugates have been found to exhibit strong to potent activity against Gram-positive and Gram-negative bacteria and fungi, with mechanisms of action attributed to bacterial membrane damage and/or membrane depolarization [12,14,20]. The mechanism of membrane disruption has been attributed to the observed ability of some mimics to exhibit antibiotic enhancing properties [22,23]. Any future exploitation of these bioactive molecules will require selectivity towards bacterial versus mammalian membranes–there are unfortunately several reports of cytotoxicity and/or hemolytic activities exhibited by squalamine mimics [13,15,20,23].

The structure of squalamine (**1**) contains the polyamine, spermidine attached at sterol position C-3. Several groups have explored bile acid-based mimics containing simple diamines or polyamines attached to the C-24 sidechain carboxylic acid, identifying examples with broad-spectrum antimicrobial activities [20,23,24,25]. An intriguing extension to these studies is a series of bile acid derivatives that are linked at C-24 by short chain diamine/triamines to form dimers, some of which exhibit antibacterial [21,22,26] and antifungal [15] properties. Majority of these dimers utilized cholic [15,21,22,26] or deoxycholic acids [15,22,26].

With only limited examples reported in the literature of C-24 amide-linked cholic acid–polyamine conjugates as squalamine mimics, we undertook a study to explore the effect of variation in the cholic acid head group, using hyodeoxycholic (**3**), ursodeoxycholic (**4**) and chenodeoxycholic (**5**) acids (Figure 2), and variation in polyamine chain length on antimicrobial properties. Compound cytotoxicity and hemolytic properties were also evaluated. Herein, we report on the synthesis and biological evaluation of this set of cholic acid-polyamine conjugates and the results of a preliminary mechanism of action evaluation.

## 2. Results and Discussion

### 2.1. Synthesis of Cholic Acid-Polyamine Conjugates

**Figure 3:**
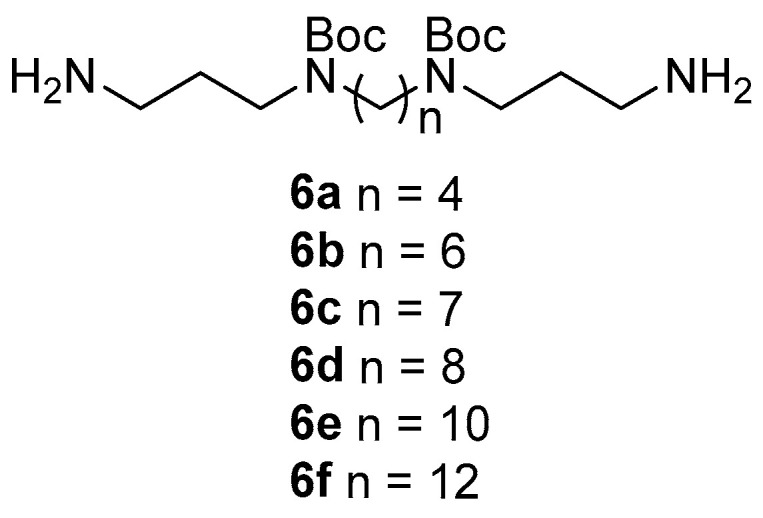
Boc-protected polyamines **6a**–**f**.

The target set of analogues required the synthesis of Boc-protected polyamine scaffolds **6a**–**f** (Figure 3), which were prepared according to literature procedures [27,28,29,30]. The polyamines chosen covered a range of overall lengths, from spermine (polyamine PA-3-4-3) through to the longer chain length PA-3-12-3 variant. The set was chosen to allow exploration of the effect of chain length, lipophilicity, and positioning of positive charges on antimicrobial and cytotoxicity/hemolytic properties.

**Scheme 1:**
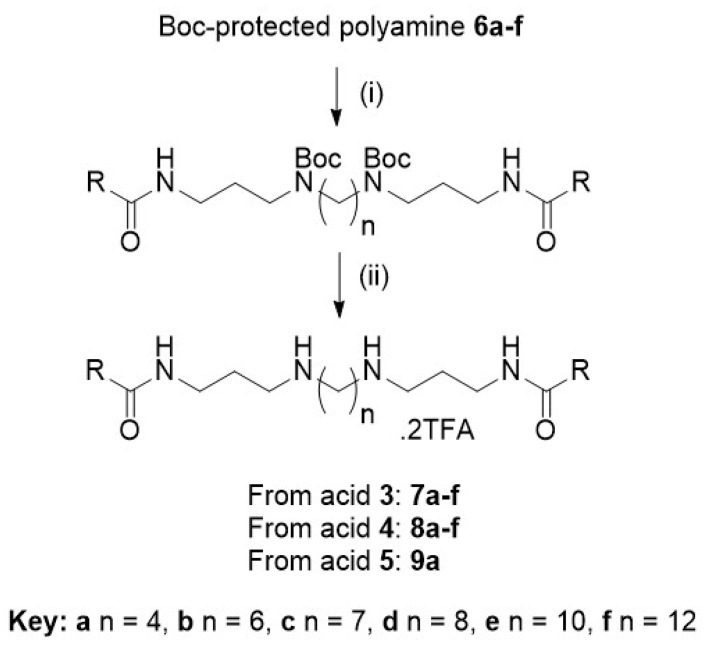
General method for the synthesis of target polyamine analogues **7**–**9**. *Reagents and conditions:* (i) for **7a**–**f**: hyodeoxycholic acid (2.0 equiv.), Boc-protected polyamine (**6a**–**f**) (1.0 equiv.), PyBOP (2.2 equiv.), DIPEA (6 equiv.), r.t., N_2_, 18 h, (yields 12–96%) or for **8a**–**f** and **9a**: carboxylic acid **4** or **5** (2.0 equiv.), Boc-protected polyamine (**6a**–**f**) (1.0 equiv.), HOBt (1.0 equiv.), DIPEA (4.0 equiv.), 0 °C, N_2_, 10 min. then EDC·HCl (3.0 equiv.), r.t., N_2_, 18 h (yields 71–92%); (ii) TFA (0.2 mL), CH_2_Cl_2_ (2 mL), r.t., 2 h (yields 59–98%).

**Figure 4:**
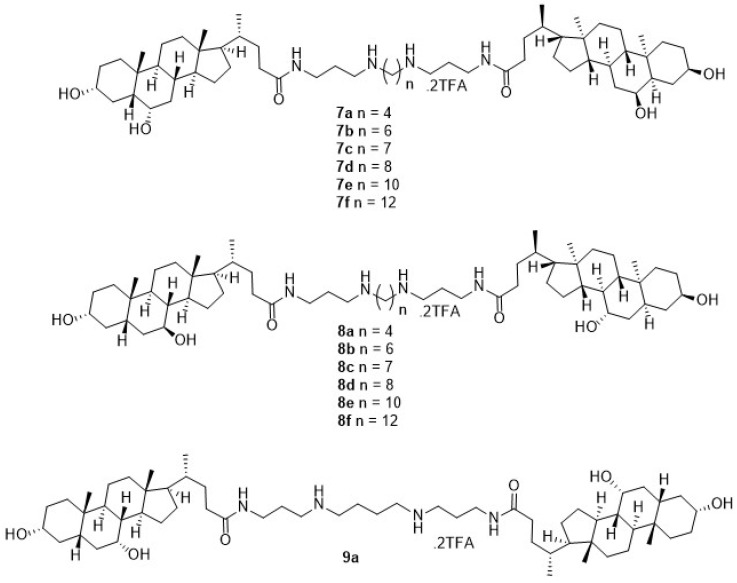
HDCA (**7a**–**f**), UDCA (**8a**–**f**) and CDCA (**9a**) analogues.

Reaction of cholic acids **3**–**5** with Boc-protected polyamines **6a**–**f** utilized coupling reagents PyBOP (benzotriazol-1-yloxytripyrrolidinophosphonium hexafluorophosphate) or EDC·HCl/HOBt (1-ethyl-3-(3-dimethylaminopropyl)carbodiimide, hydroxybenzotriazole) in anhydrous DMF, afforded coupled products which were subsequently deprotected (2,2,2-trifluoroacetic acid (TFA) in CH_2_Cl_2_) to yield the target compounds as their di-TFA salts (Scheme 1). While the preparation of conjugates **7a**–**f** (HDCA) and **8a**–**f** (UDCA) proceeded smoothly, the corresponding CDCA analogues were found to be difficult to purify, resulting in the successful synthesis, purification, and characterization of just one example (**9a**) (Figure 4).

### 2.2. Antimicrobial Activities

The antimicrobial activities of compounds **7a**–**f, 8a**–**f** and **9a** were determined against a panel of Gram-positive (methicillin-resistant *Staphylococcus aureus* (MRSA)) and Gram-negative (*Escherichia coli, Pseudomonas aeruginosa, Klebsiella pneumoniae* and *Acinetobacter baumannii*) bacteria and two fungal strains (*Candida albicans* and *Cryptococcus neoformans*) (Table 1). The overall trend observed for the compounds was growth inhibition of the Gram-positive bacteria MRSA and the fungal pathogens and limited to no activity towards the Gram-negative bacteria. The hyodeoxycholic acid (HDCA) analogues **7a**–**f** exhibited good to excellent activities against MRSA (MIC ≤ 0.19 to 3–4 μM) and *C. neoformans* (MIC ≤ 0.19 to 0.8 μM) and with the long polyamine chain variants **7e** and **7f** also exhibiting excellent activity against *C. albicans* (MIC ≤ 0.20 μM). In contrast, the ursodeoxycholic acid (UDCA) analogues **8a**–**f**, while exhibiting similar MRSA levels of activity to the HDCA series, were typically less active as antifungals. The notable exception was the longest polyamine chain variant, **8f**, which was identified as potently active against both MRSA and *C. neoformans* (MIC ≤ 0.19 μM). The single example of a chenodeoxycholic acid-polyamine conjugate (**9a**) exhibited a slightly different spectrum of antimicrobial activity compared to the other corresponding spermine derivatives **7a** and **8a**, with potent growth inhibition observed against MRSA and both fungal pathogens (MIC ≤ 0.21 μM) and was the only analogue tested that exhibited some degree of growth inhibition against a Gram-negative bacterium (MIC 6.8 μM against *E. coli*). What is notable in these results is the variation of activity, and the spectrum of activity against different microorganisms between these sets of cholic acid analogues, arising from differences in the position and stereochemistry of hydroxyl group substitution at C-6 or C-7. In the same biological assays, squalamine exhibited strong growth inhibition of the Gram-positive bacteria MRSA and Gram-negative organisms *E. coli*, *K. pneumoniae* and *A. baumannii* (MIC ≤ 0.28 μM) and weak to moderate activity towards the fungi *C. albicans* and *C. neoformans*.

### 2.3. Cytotoxic and Hemolytic Activities

As noted in a number of studies, amphipathic squalamine mimics can exhibit varying degrees of cytotoxicity and/or hemolytic properties. Cytotoxicity towards HEK293 (human kidney epithelial cell line, IC_50_) and hemolytic activity against human red blood cells (HC_10_) were determined for compounds **7**–**9** (Table 2). While the HDCA analogues **7a**–**f** were devoid of cytotoxicity and hemolytic properties at the top dose tested (32 μg/mL) and CDCA analogue **9a** exhibited low levels of toxicity (IC_50_ and HC_10_ 27 μM), the UDCA analogues **8a**–**f** exhibited hemolytic properties with **8f** also exhibiting cytotoxic properties. These results identified the HDCA series of analogues **7a**–**f** as being selective for biological activity towards microorganisms versus mammalian cells. A similar lack of toxicity indicators was also observed for squalamine.

### 2.4. Real Time Growth Inhibition Curves and Determination of Bactericidal Activity

**Figure 5:**
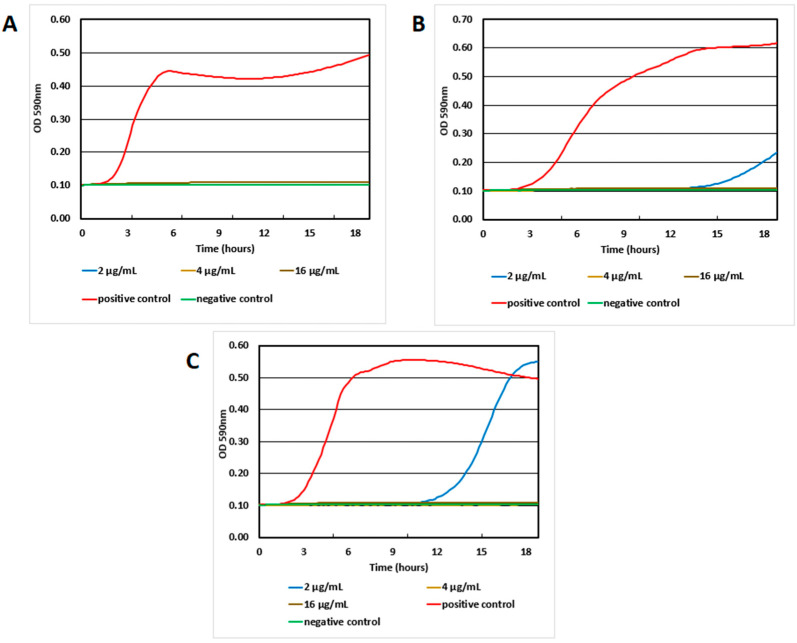
Bacterial growth inhibition exhibited by **7e** against (**A**) *S. aureus* ATCC 25923, (**B**) *S. aureus* MRSA (CF-Marseille); (**C**) *Bacillus cereus* ATCC 11778 with different concentrations. Positive control was bacteria only and negative control was media only.

The antibacterial activity of **7e** against *S. aureus* ATCC 25923, *S. aureus* MRSA (CF-Marseille) [31] and *Bacillus cereus* ATCC 11778 in liquid brain heart infusion (BHI) media were evaluated by measuring optical density (at 590 nm) after culturing from 1 to 19 h. The cholic acid-polyamine conjugate inhibited all three strains at 3.2 μM (4 µg/mL) and 12.7 μM (16 µg/mL) concentration, whereas at the lowest tested concentration, 1.6 μM (2 µg/mL), *B. cereus* exhibited growth after 10 h and *S. aureus* after 13 h (Figure 5). Classical microdilution methodology determined MIC values of **7e** towards these three microorganisms of 1.6 μM (2 μg/mL), 3.2 μM (4 μg/mL) and 3.2 μM (4 μg/mL), respectively, with the values matching those observed at 18 h in the real time growth inhibition curve plots. The same values were observed for the minimum bactericidal concentration (MBC) for **7e** against the three organisms, identifying the cholic acid-polyamine conjugate as being bactericidal.

### 2.5. Membrane Perturbation–ATP Release

**Figure 6:**
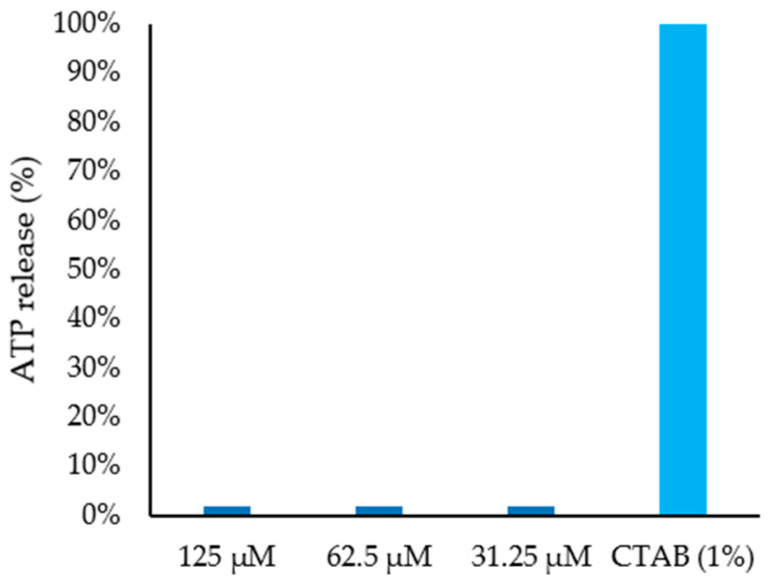
ATP release from *S. aureus* when treated with cholic acid derivative **7e** with cetyltrimethylammonium bromide (CTAB) (1%) as the positive control.

Previous studies have noted the ability of squalamine to induce rapid loss of intracellular ATP [5,7]. Using the same bioluminescence method, the ability of HDCA analogue **7e** to disrupt the membrane of *S. aureus* was investigated, with the detection of enhanced levels of extracellular ATP used as a reporter reflecting the permeabilizing effect of the compound. CTAB positive control dramatically disrupted the *S. aureus* membrane after 2 min, leading to observation of pronounced levels of fluorescence (Figure 6). In direct contrast, however, was the inability of **7e** to induce ATP release, even when examined over a 30 min period after compound exposure. This was a somewhat surprising result, suggesting that the mechanism of anti-*Staphylococcus* action of **7e** does not rely upon targeting the integrity of the bacterial membrane.

### 2.6. Antibiotic Enhancing Activities

Squalamine and some mimics can enhance the activity of antibiotics towards Gram-negative bacteria [9,21,22]. A sub-set of the HDCA analogues (**7a**, **7c**, **7d**, **7e**) were tested for the ability to enhance the antibiotic activity of doxycycline against *P. aeruginosa* ATCC 27853 and of erythromycin against *E. coli* ATCC 25922. No enhancement was observed for any of the compounds.

## 3. Materials and Methods

### 3.1. Chemical Synthesis General Methods

Mass spectra were recorded using a MicrOTOF-QII mass spectrometer (Bruker Daltonics, Bremen, Germany) coupled with a KD Scientific syringe pump, with analysis using Bruker Compass DataAnalysis v 4.1 software. Infrared spectra were recorded on a Perkin Elmer Spectrum 100 Fourier Transform infrared spectrometer (Waltham, MA, USA) equipped with a universal ATR accessory. Optical rotations were obtained with a Rudolph Analytical (Hackettstown, NJ, USA) Autopol IV automatic polarimeter using a 0.1 dm cell (concentration units of g/100 mL). All NMR spectra were recorded using a Bruker (Karlsruhe, Germany) Avance 400 spectrometer operating at 400.13 MHz for ^1^H nuclei and 100.62 MHz for ^13^C nuclei. Chemical shifts are expressed in parts per million (ppm) relative to the solvent peaks (DMSO-*d*_6_: ^1^H 2.50, ^13^C 39.52 ppm). Assignments are based on 1- and 2-dimensional NMR experiments and analogue comparisons. Standard Bruker pulse sequences were utilized. Reversed-phase flash column chromatography was carried out using LiChroPrep RP-8 (40–63 μm) (Merck Millipore, Darmstadt, Germany). Analytical thin layer chromatography (TLC) was carried out on 0.2 mm thick plates of Merck DC Kieselgel 60 RP-18 F254S plates. All solvents were of analytical grade or better and/or purified according to standard procedures. Chemical reagents used were purchased from standard chemical suppliers and used as purchased. Protected polyamines di-*tert*-butyl butane-1,4-diylbis((3-aminopropyl)carbamate) (**6a**) di-*tert*-butyl hexane-1,6-diylbis((3-aminopropyl)carbamate) (**6b**), di-*tert*-butyl heptane-1,7-diylbis((3-aminopropyl)carbamate) (**6c**), di-*tert*-butyl octane-1,8-diylbis((3-aminopropyl)carbamate) (**6d**), di-*tert*-butyl decane-1,10-diylbis((3-aminopropyl)carbamate) (**6e**), and di-*tert*-butyl dodecane-1,12-diylbis((3-aminopropyl)carbamate) (**6f**) were synthesized using literature procedures [27,28,29,30].

#### 3.1.1. General Procedure A: Amide Bond Formation for Hyodeoxycholic Acid Derivatives **7a**–**f**

To a solution of hyodeoxycholic acid (0.050 g, 0.127 mmol) in dry DMF (1 mL) was added Boc-protected polyamine (0.063 mmol) in dry DMF (1 mL), PyBop (0.073 g, 0.140 mmol) and DIPEA (0.067 mL, 0.382 mmol). The solution was allowed to stir for 18 h at rt under N_2_ atmosphere. The resulting solution was added to EtOAc (20 mL) and was washed with H_2_O (5 × 20 mL), then dried under reduced pressure and the crude product was subjected to diol-bonded silica column chromatography (CH_2_Cl_2_/MeOH, 80:20→90:10) to yield the desired Boc-protected hyodeoxycholic acid derivative.

#### 3.1.2. General Procedure B: Amide Bond Formation for Cholic Acid Derivatives **8a**–**f** and **9a**

To an ice-cold solution of the appropriate cholic acid (2 equiv.) in dry DMF (1 mL) was added Boc-protected polyamine (1 equiv.), HOBt (1 equiv.) and DIPEA (4 equiv.). The solution was stirred for 10 min at 0 °C under N_2_ atmosphere. EDC·HCl (3 equiv.) in dry DMF (1 mL) was added to the solution and the resulting mixture was left to stir for 18 h at rt under N_2_ atmosphere. The resulting solution was added to EtOAc (20 mL) and washed with H_2_O (2 × 20 mL). The organic layer was dried under reduced pressure and the crude product was purified with silica gel column chromatography (CH_2_Cl_2_/MeOH, 80:20→90:10) to afford the desired Boc-protected product.

#### 3.1.3. General Procedure C: Boc Deprotection

A solution of the *tert*-butyl-carbamate derivative was stirred in CH_2_Cl_2_ (2 mL) with TFA (0.2 mL) at room temperature under N_2_ for 2 h, then dried under reduced pressure. The crude product was purified using C_8_ reversed-phase column chromatography (MeOH (+0.05% TFA):H_2_O (+0.05% TFA), 50:50→100:0) to afford the product as the di-TFA salt.

#### 3.1.4. *N*^1^,*N*^4^-Bis(3-((*R*)-4-((3*R*,5*R*,6*S*,8*S*,9*S*,10*R*,13*R*,14*S*,17*R*)-3,6-dihydroxy-10,13-dimethylhexadecahydro-1*H*-cyclopenta[*a*]phenanthren-17-yl)pentanamido)propyl)butane-1,4-diaminium 2,2,2-trifluoroacetate (**7a**)

Following general procedure A, reaction of hyodeoxycholic acid (0.050 g, 0.127 mmol) with di-*tert*-butyl butane-1,4-diylbis((3-aminopropyl)carbamate) (**6a**) (0.026 g, 0.0637 mmol), PyBop (0.073 g, 0.140 mmol) and DIPEA (0.067 mL, 0.382 mmol) in DMF (2 mL) afforded di-*tert*-butyl butane-1,4-diylbis((3-((*R*)-4-((3*R*,5*R*,6*S*,8*S*,9*S*,10*R*,13*R*,14*S*,17*R*)-3,6-dihydroxy-10,13-dimethylhexadecahydro-1*H*-cyclopenta[*a*]phenanthren-17-yl)pentanamido)propyl)carbamate) (0.056 g, 76%) as a clear colorless oil. Following general procedure C, a sub-sample of the product (0.047 g, 0.0408 mmol) was reacted with TFA in CH_2_Cl_2_ to afford, after chromatography, the di-TFA salt **7a** (0.034 g, 71%) as a pale-yellow oil. [α${]}_{D}^{20.3}$ = +3 (*c* = 0.1, MeOH); R*_f_* (RP-18, 10% *aq*. HCl:MeOH 1:3) 0.17; IR (ATR) ν_max_ 3339, 2941, 1635, 1455, 1202, 1139, 1027 cm^−1^; ^1^H NMR (DMSO-*d*_6_, 400 MHz) δ 8.63 (4H, br s, NH_2_-29), 8.01 (2H, t, *J* = 5.7 Hz, NH-25), 3.84–3.79 (2H, m, H-6), 3.35–3.27 (2H, m, H-3), 3.09 (4H, q, *J* = 6.3 Hz, H_2_-26), 2.90–2.87 (8H, m, H_2_-28, H_2_-30), 2.11–2.08 (2H, m, H_2_-23b), 1.99–-1.95 (2H, m, H_2_-23a), 1.92–0.93 (56H, m, H_2_-1, H_2_-2, H_2_-4, H-5, H_2_-7, H-8, H-9, H_2_-11, H_2_-12, H-14, H_2_-15, H_2_-16, H-17, H-20, H_2_-22, H_2_-27, H_2_-31), 0.87 (6H, d, *J* = 6.4 Hz, H_3_-21), 0.83 (6H, s, H_3_-19), 0.59 (6H, s, H_3_-18); ^13^C NMR (DMSO-*d*_6_, 100 MHz) δ 173.2 (C-24), 70.0 (C-3), 65.9 (C-6), 55.9 (C-14), 55.5 (C-17), 48.3 (C-5), 46.1 (C-30), 44.6 (C-28), 42.4 (C-13), 39.5 (C-9, obscured by solvent), 39.4 (C-12, obscured by solvent), 35.5, 35.4 (C-1, C-26), 35.0, 34.9 (C-7, C-8, C-10), 34.4 (C-20), 32.3 (C-23), 31.5 (C-22), 30.3 (C-4), 29.3 (C-2), 27.7 (C-16), 26.1 (C-27), 23.9 (C-15), 23.6 (C-19), 22.7 (C-31), 20.4 (C-11), 18.3 (C-21), 11.9 (C-18); (+)-HRESIMS [M + H]^+^ *m/z* 951.7873 (calcd for C_58_H_103_N_4_O_6_, 951.7872).

#### 3.1.5. *N*^1^,*N*^6^-Bis(3-((*R*)-4-((3*R*,5*R*,6*S*,8*S*,9*S*,10*R*,13*R*,14*S*,17*R*)-3,6-dihydroxy-10,13-dimethylhexadecahydro-1*H*-cyclopenta[*a*]phenanthren-17-yl)pentanamido)propyl)hexane-1,6-diaminium 2,2,2-trifluoroacetate (**7b**)

Following general procedure A, reaction of hyodeoxycholic acid (0.050 g, 0.127 mmol) with di-*tert*-butyl hexane-1,6-diylbis((3-aminopropyl)carbamate) (**6b**) (0.027 g, 0.0637 mmol), PyBop (0.073 g, 0.140 mmol) and DIPEA (0.067 mL, 0.382 mmol) in DMF (2 mL) afforded di-*tert*-butyl hexane-1,6-diylbis((3-((*R*)-4-((3*R*,5*R*,6*S*,8*S*,9*S*,10*R*,13*R*,14*S*,17*R*)-3,6-dihydroxy-10,13-dimethylhexadecahydro-1*H*-cyclopenta[*a*]phenanthren-17-yl)pentanamido)propyl)carbamate) (0.014 g, 19%) as a clear colorless oil. Following general procedure C, a sub-sample of the product (0.013 g, 0.0110 mmol) was reacted with TFA in CH_2_Cl_2_ to afford, after chromatography, the di-TFA salt **7b** (0.011 g, 83%) as a clear colorless oil. [α${]}_{D}^{21.3}$ = +4 (*c* = 0.1, MeOH); R*_f_* (RP-18, 10% *aq*. HCl:MeOH 1:3) 0.14; IR (ATR) ν_max_ 2938, 1676, 1204, 1137, 1055, 1033 cm^−1^; ^1^H NMR (DMSO-*d*_6_, 400 MHz) δ 8.42 (4H, br s, NH_2_-29), 7.98 (2H, t, *J* = 5.8 Hz, NH-25), 3.84–3.81 (2H, m, H-6), 3.32 (2H, obscured by H_2_O, H-3), 3.10 (4H, q, *J* = 6.3 Hz, H_2_-26), 2.86 (8H, br s, H_2_-28, H_2_-30), 2.11–2.07 (2H, m, H_2_-23b), 2.02–1.96 (2H, m, H_2_-23a), 1.94–0.91 (60H, m, H_2_-1, H_2_-2, H_2_-4, H-5, H_2_-7, H-8, H-9, H_2_-11, H_2_-12, H-14, H_2_-15, H_2_-16, H-17, H-20, H_2_-22, H_2_-27, H_2_-31, H_2_-32), 0.88 (6H, d, *J* = 6.5 Hz, H_3_-21), 0.83 (6H, s, H_3_-19), 0.60 (6H, s, H_3_-18); ^13^C NMR (DMSO-*d*_6_, 100 MHz) δ 173.3 (C-24), 69.9 (C-3), 65.8 (C-6), 55.9 (C-14), 55.5 (C-17), 48.2 (C-5), 46.6 (C-30), 44.6 (C-28), 42.3 (C-13), 39.4 (C-12, obscured by solvent), 39.3 (C-9, obscured by solvent), 35.5, 35.4 (C-1, C-26), 34.9, 34.8 (C-7, C-8, C-10), 34.3 (C-20), 32.2 (C-23), 31.5 (C-22), 30.3 (C-4), 29.2 (C-2), 27.7 (C-16), 26.2 (C-27), 25.5 (C-31), 25.3 (C-32), 23.8 (C-15), 23.5 (C-19), 20.4 (C-11), 18.3 (C-21), 11.9 (C-18); (+)-HRESIMS [M + H]^+^ *m/z* 979.8185 (calcd for C_60_H_107_N_4_O_6_, 979.8185).

#### 3.1.6. *N*^1^,*N*^7^-Bis(3-((*R*)-4-((3*R*,5*R*,6*S*,8*S*,9*S*,10*R*,13*R*,14*S*,17*R*)-3,6-dihydroxy-10,13-dimethylhexadecahydro-1*H*-cyclopenta[*a*]phenanthren-17-yl)pentanamido)propyl)heptane-1,7-diaminium 2,2,2-trifluoroacetate (**7c**)

Following general procedure A, reaction of hyodeoxycholic acid (0.050 g, 0.127 mmol) with di-*tert*-butyl heptane-1,7-diylbis((3-aminopropyl)carbamate) (**6c**) (0.028 g, 0.0637 mmol), PyBop (0.073 g, 0.140 mmol) and DIPEA (0.067 mL, 0.382 mmol) in DMF (2 mL) afforded di-*tert*-butyl heptane-1,7-diylbis((3-((*R*)-4-((3*R*,5*R*,6*S*,8*S*,9*S*,10*R*,13*R*,14*S*,17*R*)-3,6-dihydroxy-10,13-dimethylhexadecahydro-1*H*-cyclopenta[*a*]phenanthren-17-yl)pentanamido)propyl)carbamate) (0.061 g, 80%) as a clear oil. Following general procedure C, a sub-sample of the product (0.045 g, 0.0377 mmol) was reacted with TFA in CH_2_Cl_2_ to afford, after chromatography, the di-TFA salt **7c** (0.041, 89%) as a pale-yellow oil. [α${]}_{D}^{20.6}$= +2 (*c* = 0.1, MeOH); R*_f_* (RP-18, 10% *aq*. HCl:MeOH 1:3) 0.13; IR (ATR) ν_max_ 3361, 2931, 1675, 1202, 1135, 1038, 1026, 993 cm^−1^; ^1^H NMR (DMSO-*d*_6_, 400 MHz) δ 8.52 (4H, br s, NH_2_-29), 8.00 (2H, t, *J* = 5.8 Hz, NH-25), 3.83–3.80 (2H, m, H-6), 3.35–3.25 (4H, m, H-3, H-7), 3.10 (4H, q, *J* = 6.3 Hz, H_2_-26), 2.89–2.82 (8H, m, H_2_-28, H_2_-30), 2.15–2.07 (2H, m, H_2_-23b), 2.02–1.97 (2H, m, H-23a), 1.94–0.92 (62H, m, H_2_-1, H_2_-2, H_2_-4, H-5, H_2_-7, H-8, H-9, H_2_-11, H_2_-12, H-14, H_2_-15, H_2_-16, H-17, H-20, H_2_-22, H_2_-27, H_2_-31, H_2_-32, H_2_-33), 0.89 (6H, d, *J* = 6.5 Hz, H_3_-21), 0.87 (6H, s, H_3_-19), 0.59 (6H, s, H_3_-18); ^13^C NMR (DMSO-*d*_6_, 100 MHz) δ 173.2 (C-24), 70.0 (C-3), 65.9 (C-6), 55.9 (C-14), 55.5 (C-17), 48.3 (C-5), 46.7 (C-30), 44.6 (C-28), 42.4 (C-13), 39.4 (C-9, obscured by solvent), 39.3 (C-12, obscured by solvent), 35.6, 35.5 (C-1, C-26), 35.0 (C-7), 34.9 (C-8, C-10), 34.4 (C-20), 32.3 (C-23), 31.5 (C-22), 30.3 (C-4), 29.3 (C-2), 28.0 (C-33), 27.7 (C-16), 26.1 (C-27), 25.8 (C-32), 25.4 (C-31), 23.9 (C-15), 23.6 (C-19), 20.4 (C-11), 18.3 (C-21), 11.9 (C-18); (+)-HRESIMS [M + H]^+^ *m/z* 993.8342 (calcd for C_61_H_109_N_4_O_6_, 993.8342).

#### 3.1.7. *N*^1^,*N*^8^-Bis(3-((*R*)-4-((3*R*,5*R*,6*S*,8*S*,9*S*,10*R*,13*R*,14*S*,17*R*)-3,6-dihydroxy-10,13-dimethylhexadecahydro-1*H*-cyclopenta[*a*]phenanthren-17-yl)pentanamido)propyl)octane-1,8-diaminium 2,2,2-trifluoroacetate (**7d**)

Following general procedure A, reaction of hyodeoxycholic acid (0.050 g, 0.127 mmol) with di-*tert*-butyl octane-1,8-diylbis((3-aminopropyl)carbamate) (**6d**) (0.029 g, 0.0637 mmol), PyBop (0.073 g, 0.140 mmol) and DIPEA (0.067 mL, 0.382 mmol) in DMF (2 mL) afforded di-*tert-*butyl octane-1,8-diylbis((3-((*R*)-4-((3*R*,5*R*,6*S*,8*S*,9*S*,10*R*,13*R*,14*S*,17*R*)-3,6-dihydroxy-10,13-dimethylhexadecahydro-1*H*-cyclopenta[*a*]phenanthren-17-yl)pentanamido)propyl)carbamate) (0.074 g, 96%) as a clear colorless oil. Following general procedure C, the product (0.074 g, 0.0613 mmol) was reacted with TFA in CH_2_Cl_2_ to afford, after chromatography, the di-TFA salt **7d** (0.063 g, 83%) as a pale-yellow oil. [α${]}_{D}^{20.5}$ = +5 (*c* = 0.1, MeOH); R*_f_* (RP-18, 10% *aq*. HCl:MeOH 1:3) 0.13; IR (ATR) ν_max_ 3343, 2938, 2865, 1635, 1456, 1377, 1201, 1136, 1028, 800, 721 cm^−1^; ^1^H NMR (DMSO-*d*_6_, 400 MHz) δ 8.55 (4H, br s, NH_2_-29), 8.01 (2H, t, *J* = 5.5 Hz, NH-25), 3.84–3.79 (2H, m, H-6), 3.35–3.28 (2H, m, H-3), 3.09 (4H, q, *J* = 6.0 Hz, H_2_-26), 2.85 (8H, br s, H_2_-28, H_2_-30), 2.14–2.07 (2H, m, H_2_-23b), 2.01–1.96 (2H, m, H_2_-23a), 1.94–0.91 (64H, m, H_2_-1, H_2_-2, H_2_-4, H-5, H_2_-7, H-8, H-9, H_2_-11, H_2_-12, H-14, H_2_-15, H_2_-16, H-17, H-20, H_2_-22, H_2_-27, H_2_-31, H_2_-32, H_2_-33), 0.87 (6H, d, *J* = 6.2 Hz, H_3_-21), 0.83 (6H, s, H_3_-19), 0.59 (6H, s, H_3_-18); ^13^C NMR (DMSO-*d*_6_, 100 MHz) δ 173.4 (C-24), 70.1 (C-3), 66.0 (C-6), 56.0 (C-14), 55.6 (C-17), 48.3 (C-5), 46.8 (C-30), 44.7 (C-28), 42.4 (C-13), 39.5 (C-12, obscured by solvent), 39.4 (C-9, obscured by solvent), 35.6, 35.5 (C-1, C-26), 35.0, 34.9 (C-7, C-8, C-10), 34.4 (C-20), 32.3 (C-23), 31.6 (C-22), 30.4 (C-4), 29.3 (C-2), 28.4 (C-33), 27.8 (C-16), 26.2 (C-27), 25.9 (C-32), 25.5 (C-31), 23.9 (C-15), 23.6 (C-19), 20.5 (C-11), 18.3 (C-21), 11.9 (C-18); (+)-HRESIMS [M + 2H]^2+^ *m/z* 504.4286 (calcd for C_62_H_112_N_4_O_6_, 504.4285).

#### 3.1.8. *N*^1^,*N*^10^-Bis(3-((*R*)-4-((3*R*,5*R*,6*S*,8*S*,9*S*,10*R*,13*R*,14*S*,17*R*)-3,6-dihydroxy-10,13-dimethylhexadecahydro-1*H*-cyclopenta[*a*]phenanthren-17-yl)pentanamido)propyl)decane-1,10-diaminium 2,2,2-trifluoroacetate (**7e**)

Following general procedure A, reaction of hyodeoxycholic acid (0.050 g, 0.127 mmol) with di-*tert*-butyl decane-1,10-diylbis((3-aminopropyl)carbamate) (**6e**) (0.031 g, 0.0637 mmol), PyBop (0.073 g, 0.140 mmol) and DIPEA (0.067 mL, 0.382 mmol) in DMF (2 mL) afforded di-*tert*-butyl decane-1,10-diylbis((3-((*R*)-4-((3*R*,5*R*,6*S*,8*S*,9*S*,10*R*,13*R*,14*S*,17*R*)-3,6-dihydroxy-10,13-dimethylhexadecahydro-1*H*-cyclopenta[*a*]phenanthren-17-yl)pentanamido)propyl)carbamate) (0.065 g, 83%) as a clear colorless oil. Following general procedure C, a sub-sample of the product (0.013 g, 0.0105 mmol) was reacted with TFA in CH_2_Cl_2_ to afford, after chromatography, the di-TFA salt **7e** (0.011 g, 83%) as a pale-yellow oil. [α${]}_{D}^{20.2}$ = +4 (*c* = 0.1, MeOH); R*_f_* (RP-18, 10% *aq*. HCl:MeOH 1:3) 0.10; IR (ATR) ν_max_ 3333, 2935, 2864, 1674, 1456, 1202, 1137, 1028, 721 cm^−1^; ^1^H NMR (DMSO-*d*_6_, 400 MHz) δ 8.40 (4H, br s, NH_2_-29), 8.00 (2H, t, *J* = 5.5 Hz, NH-25), 3.84–3.80 (2H, m, H-6), 3.34–3.29 (2H, m, H-3), 3.10 (4H, q, *J* = 6.1 Hz, H_2_-26), 2.85 (8H, br s, H_2_-28, H_2_-30), 2.14–2.07 (2H, m, H_2_-23b), 2.02–1.97 (2H, m, H_2_-23a), 1.94–0.92 (68H, m, H_2_-1, H_2_-2, H_2_-4, H-5, H_2_-7, H-8, H-9, H_2_-11, H_2_-12, H-14, H_2_-15, H_2_-16, H-17, H-20, H_2_-22, H_2_-27, H_2_-31, H_2_-32_,_ H_2_-33, H_2_-34), 0.88 (6H, d, *J* = 6.1 Hz, H_3_-21), 0.83 (6H, s, H_3_-19), 0.60 (6H, s, H_3_-18); ^13^C NMR (DMSO-*d*_6_, 100 MHz) δ 173.3 (C-24), 70.0 (C-3), 65.9 (C-6), 55.9 (C-14), 55.5 (C-17), 48.2 (C-5), 46.8 (C-30), 44.5 (C-28), 42.4 (C-13), 39.5 (C-12 and C-9, obscured by solvent), 35.5, 35.4 (C-1, C-26), 35.0, 34.9 (C-7, C-8, C-10), 34.3 (C-20), 32.3 (C-23), 31.5 (C-22), 30.3 (C-4), 29.3 (C-2), 28.8 (C-34), 28.6 (C-33), 27.7 (C-16), 26.2 (C-27), 25.9 (C-32), 25.5 (C-31), 23.9 (C-15), 23.5 (C-19), 20.4 (C-11), 18.3 (C-21), 11.9 (C-18); (+)-HRESIMS [M + 2H]^2+^ *m/z* 518.4441 (calcd for C_64_H_116_N_4_O_6_, 518.4442).

#### 3.1.9. *N*^1^,*N*^12^-Bis(3-((*R*)-4-((3*R*,5*R*,6*S*,8*S*,9*S*,10*R*,13*R*,14*S*,17*R*)-3,6-dihydroxy-10,13-dimethylhexadecahydro-1*H*-cyclopenta[*a*]phenanthren-17-yl)pentanamido)propyl)dodecane-1,12-diaminium 2,2,2-trifluoroacetate (**7f**)

Following general procedure A, reaction of hyodeoxycholic acid (0.050 g, 0.127 mmol) with di-*tert*-butyl dodecane-1,12-diylbis((3-aminopropyl)carbamate) (**6f**) (0.033 g, 0.0637 mmol), PyBop (0.073 g, 0.140 mmol) and DIPEA (0.067 mL, 0.382 mmol) in DMF (2 mL) afforded di-*tert*-butyl dodecane-1,12-diylbis((3-((*R*)-4-((3*R*,5*R*,6*S*,8*S*,9*S*,10*R*,13*R*,14*S*,17*R*)-3,6-dihydroxy-10,13-dimethylhexadecahydro-1*H*-cyclopenta[*a*]phenanthren-17-yl)pentanamido)propyl)carbamate) (0.010 g, 12%) as a clear colorless oil. Following general procedure C, the product (0.010 g, 0.00791 mmol) was reacted with TFA in CH_2_Cl_2_ to afford, after chromatography, the di-TFA salt **7f** (0.008 g, 78%) as a pale-yellow oil. [α${]}_{D}^{19.5}$= +5 (*c* = 0.1, MeOH); R*_f_* (RP-18, 10% *aq*. HCl:MeOH 1:3) 0.09; IR (ATR) ν_max_ 3340, 2932, 1643, 1203, 1138, 1039, 1026, 988 cm^−1^; ^1^H NMR (DMSO-*d*_6_, 400 MHz) δ 8.33 (4H, br s, NH_2_-29), 8.00 (2H, t, *J* = 5.8 Hz, NH-25), 3.84–3.79 (2H, m, H-6), 3.35–3.29 (2H, m, H-3), 3.10 (4H, q, *J* = 6.3 Hz, H_2_-26), 2.85 (8H, br s, H_2_-28, H_2_-30), 2.15–2.08 (2H, m, H_2_-23b), 2.02–1.97 (2H, m, H_2_-23a), 1.95–0.95 (72H, m, H_2_-1, H_2_-2, H_2_-4, H-5, H_2_-7, H-8, H-9, H_2_-11, H_2_-12, H-14, H_2_-15, H_2_-16, H-17, H-20, H_2_-22, H_2_-27, H_2_-31, H_2_-32_,_ H_2_-33, H_2_-34, H_2_-35), 0.88 (6H, d, *J* = 6.5 Hz, H_3_-21), 0.83 (6H, s, H_3_-19), 0.60 (6H, s, H_3_-18); ^13^C NMR (DMSO-*d*_6_, 100 MHz) δ 173.3 (C-24), 70.0 (C-3), 65.9 (C-6), 55.9 (C-14), 55.5 (C-17), 48.2 (C-5), 46.8 (C-30), 44.5 (C-28), 42.4 (C-13), 39.6 (C-12, obscured by solvent), 39.2 (C-9, obscured by solvent), 35.5, 35.4 (C-1, C-26), 34.94, 34.86 (C-7, C-8, C-10), 34.3 (C-20), 32.3 (C-23), 31.5 (C-22), 30.3 (C-4), 29.2 (C-2), 29.0 (C-35), 28.9 (C-34), 28.6 (C-33), 27.7 (C-16), 26.2 (C-27), 25.9 (C-32), 25.5 (C-31), 23.9 (C-15), 23.5 (C-19), 20.4 (C-11), 18.3 (C-21), 11.9 (C-18); (+)-HRESIMS [M + H]^+^ *m/z* 1063.9127 (calcd for C_66_H_119_N_4_O_6_, 1063.9124).

#### 3.1.10. *N*^1^,*N*^4^-Bis(3-((*R*)-4-((3*R*,5*S*,7*S*,8*R*,9*S*,10*S*,13*R*,14*S*,17*R*)-3,7-dihydroxy-10,13-dimethylhexadecahydro-1*H*-cyclopenta[*a*]phenanthren-17-yl)pentanamido)propyl)butane-1,4-diaminium 2,2,2-trifluoroacetate (**8a**)

Following general procedure B, ursodeoxycholic acid (0.045 g, 0.115 mmol) was reacted with di-*tert*-butyl butane-1,4-diylbis((3-aminopropyl)carbamate) (**6a**) (0.023 g, 0.0573 mmol), EDC·HCl (0.033 g, 0.172 mmol), HOBt (0.008 g, 0.0573 mmol) and DIPEA (0.040 mL, 0.229 mmol) to afford di-*tert*-butyl butane-1,4-diylbis((3-((*R*)-4-((3*R*,5*S*,7*S*,8*R*,9*S*,10*S*,13*R*,14*S*,17*R*)-3,7-dihydroxy-10,13-dimethylhexadecahydro-1*H*-cyclopenta[*a*]phenanthren-17-yl)pentanamido)propyl)carbamate) (0.061 g, 92%) as a clear colorless oil. Following general procedure C, a sub-sample of the product (0.005 g, 0.00434 mmol) was reacted with TFA in CH_2_Cl_2_ to afford, after chromatography, the di-TFA salt **8a** (0.005 g, 98%) as a pale-yellow oil. [α${]}_{D}^{19.1}$ = +24 (*c* = 0.1, MeOH); R*_f_* (RP-18, 10% *aq*. HCl:MeOH 1:3) 0.26; IR (ATR) ν_max_ 3328, 1638, 1202, 1038, 1027 cm^−1^; ^1^H NMR (DMSO-*d*_6_, 400 MHz) δ 8.42 (4H, br s, NH_2_-29), 7.99 (2H, t, *J* = 5.5 Hz, NH-25), 3.39–3.28 (4H, m, H-3, H-7), 3.11 (4H, q, *J* = 6.2 Hz, H_2_-26), 2.92–2.88 (8H, m, H_2_-28, H_2_-30), 2.09–0.83 (72H, m, H_2_-1, H_2_-2, H_2_-4, H-5, H_2_-6, H-8, H-9, H_2_-11, H_2_-12, H-14, H_2_-15, H_2_-16, H-17, H_3_-19, H-20, H_3_-21, H_2_-22, H_2_-23, H_2_-27, H_2_-31), 0.61 (6H, s, H_3_-18); ^13^C NMR (DMSO-*d*_6_, 100 MHz) δ 173.3 (C-24), 69.7 (C-3), 69.5 (C-7), 55.9 (C-14), 54.7 (C-17), 46.1 (C-30), 44.6 (C-28), 43.1, 43.0 (C-8, C-13), 42.2 (C-5), 39.2 (C-12, obscured by solvent), 38.8 (C-10), 37.7 (C-4), 37.3 (C-6), 35.6 (C-26), 35.0, 34.9 (C-9, C-15, C-20), 33.8 (C-1), 32.4 (C-23), 31.7 (C-22), 30.2 (C-2), 28.2 (C-16), 26.1 (C-27), 23.3 (C-19), 22.7 (C-31), 20.9 (C-11), 18.4 (C-21), 12.0 (C-18); (+)-HRESIMS [M + H]^+^ *m/z* 951.7873 (calcd for C_58_H_103_N_4_O_6_, 951.7872).

#### 3.1.11. *N*^1^,*N*^6^-Bis(3-((*R*)-4-((3*R*,5*S*,7*S*,8*R*,9*S*,10*S*,13*R*,14*S*,17*R*)-3,7-dihydroxy-10,13-dimethylhexadecahydro-1*H*-cyclopenta[*a*]phenanthren-17-yl)pentanamido)propyl)hexane-1,6-diaminium 2,2,2-trifluoroacetate (**8b**)

Following general procedure B, ursodeoxycholic acid (0.050 g, 0.127 mmol) was reacted with di-*tert*-butyl hexane-1,6-diylbis((3-aminopropyl)carbamate) (**6b**) (0.027 g, 0.0637 mmol), EDC·HCl (0.037 g, 0.191 mmol), HOBt (0.009 g, 0.0637 mmol) and DIPEA (0.044 mL, 0.255 mmol) affording di-*tert*-butyl hexane-1,6-diylbis((3-((*R*)-4-((3*R*,5*S*,7*S*,8*R*,9*S*,10*S*,13*R*,14*S*,17*R*)-3,7-dihydroxy-10,13-dimethylhexadecahydro-1*H*-cyclopenta[*a*]phenanthren-17-yl)pentanamido)propyl)carbamate) (0.061 g, 81%) as a clear colorless oil. Following general procedure C, a sub-sample of the product (0.043 g, 0.0364 mmol) was reacted with TFA in CH_2_Cl_2_ to afford, after chromatography, the di-TFA salt **8b** (0.030 g, 68%) as a pale-yellow oil. [α${]}_{D}^{20.1}$ = +20 (*c* = 0.1, MeOH); R*_f_* (RP-18, 10% *aq*. HCl:MeOH 1:3) 0.23; IR (ATR) ν_max_ 3308, 2935, 2866, 1637, 1456, 1202, 1138, 1050 cm^−1^; ^1^H NMR (DMSO-*d*_6_, 400 MHz) δ 8.45 (4H, br s, NH_2_-29), 7.98 (2H, t, *J* = 5.7 Hz, NH-25), 4.46 (2H, br s, OH-3 or OH-7), 3.87 (2H, d, *J* = 6.3 Hz, OH-3 or OH-7), 3.32 (4H, obscured by water, H-3, H-7), 3.10 (4H, q, *J* = 6.3 Hz, H_2_-26), 2.86 (8H, br s, H_2_-28, H_2_-30), 2.15–2.07 (2H, m, H_2_-23b), 2.02–1.97 (2H, m, H_2_-23a), 1.96–0.91 (60H, m, H_2_-1, H_2_-2, H_2_-4, H-5, H_2_-6, H-8, H-9, H_2_-11, H_2_-12, H-14, H_2_-15, H_2_-16, H-17, H-20, H_2_-22, H_2_-27, H_2_-31, H_2_-32), 0.89 (6H, d, *J* = 6.5 Hz, H_3_-21), 0.87 (6H, s, H_3_-19), 0.61 (6H, s, H_3_-18); ^13^C NMR (DMSO-*d*_6_, 100 MHz) δ 173.3 (C-24), 69.7 (C-3), 69.5 (C-7), 55.9 (C-14), 54.7 (C-17), 46.6 (C-30), 44.6 (C-28), 43.1, 43.0 (C-8, C-13), 42.1 (C-5), 39.2 (C-12, obscured by solvent), 38.7 (C-10), 37.7 (C-4), 37.2 (C-6), 35.5 (C-26), 35.0 (C-15), 34.9, 34.8 (C-9, C-20), 33.7 (C-1), 32.3 (C-23), 31.6 (C-22), 30.2 (C-2), 28.2 (C-16), 26.2 (C-27), 25.5 (C-32), 25.3 (C-31), 23.3 (C-19), 20.8 (C-11), 18.4 (C-21), 12.0 (C-18); (+)-HRESIMS [M + 2H]^2+^ *m/z* 490.4129 (calcd for C_60_H_108_N_4_O_6_, 490.4129).

#### 3.1.12. *N*^1^,*N*^7^-Bis(3-((*R*)-4-((3*R*,5*S*,7*S*,8*R*,9*S*,10*S*,13*R*,14*S*,17*R*)-3,7-dihydroxy-10,13-dimethylhexadecahydro-1*H*-cyclopenta[*a*]phenanthren-17-yl)pentanamido)propyl)heptane-1,7-diaminium 2,2,2-trifluoroacetate (**8c**)

Following general procedure B, ursodeoxycholic acid (0.050 g, 0.127 mmol) was reacted with di-*tert*-butyl heptane-1,7-diylbis((3-aminopropyl)carbamate) (**6c**) (0.028 g, 0.0637 mmol), EDC·HCl (0.037 g, 0.191 mmol), HOBt (0.009 g, 0.0637 mmol) and DIPEA (0.044 mL, 0.255 mmol) affording di-*tert*-butyl heptane-1,7-diylbis((3-((*R*)-4-((3*R*,5*S*,7*S*,8*R*,9*S*,10*S*,13*R*,14*S*,17*R*)-3,7-dihydroxy-10,13-dimethylhexadecahydro-1*H*-cyclopenta[*a*]phenanthren-17-yl)pentanamido)propyl)carbamate) (0.054 g, 71%) as a white foam. Following general procedure C, a sub-sample of the product (0.020 g, 0.0168 mmol) was reacted with TFA in CH_2_Cl_2_ to afford, after chromatography, the di-TFA salt **8c** (0.012 g, 59%) as a pale-yellow oil. [α${]}_{D}^{19.6}$ = +36 (*c* = 0.1, MeOH); R*_f_* (RP-18, 10% *aq*. HCl:MeOH 1:3) 0.23; IR (ATR) ν_max_ 3345, 2937, 2867, 1635, 1455, 1202, 1138, 1052, 1033, 1013 cm^−1^; ^1^H NMR (DMSO-*d*_6_, 400 MHz) δ 8.69 (4H, br s, NH_2_-29), 8.01 (2H, t, *J* = 5.7 Hz, NH-25), 3.31–3.25 (4H, m, H-3, H-7), 3.10 (4H, q, *J* = 6.3 Hz, H_2_-26), 2.84 (8H, br s, H_2_-28, H_2_-30), 2.15–2.07 (2H, m, H_2_-23b), 2.02–1.97 (2H, m, H_2_-23a), 1.96–0.91 (62H, m, H_2_-1, H_2_-2, H_2_-4, H-5, H_2_-6, H-8, H-9, H_2_-11, H_2_-12, H-14, H_2_-15, H_2_-16, H-17, H-20, H_2_-22, H_2_-27, H_2_-31, H_2_-32, H_2_-33), 0.89 (6H, d, *J* = 6.5 Hz, H_3_-21), 0.87 (6H, s, H_3_-19), 0.61 (6H, s, H_3_-18); ^13^C NMR (DMSO-*d*_6_, 100 MHz) δ 173.1 (C-24), 69.7 (C-3), 69.4 (C-7), 55.9 (C-14), 54.7 (C-17), 46.6 (C-30), 44.5 (C-28), 43.1, 43.0 (C-8, C-13), 42.1 (C-5), 39.2 (C-12, obscured by solvent), 38.7 (C-10), 37.7 (C-4), 37.2 (C-6), 35.5 (C-26), 35.0, 34.8 (C-9, C-15, C-20), 33.7 (C-1), 32.3 (C-23), 31.6 (C-22), 30.2 (C-2), 28.2 (C-16), 27.9 (C-33), 26.0 (C-27), 25.7 (C-32), 25.3 (C-31), 23.3 (C-19), 20.8 (C-11), 18.4 (C-21), 12.0 (C-18); (+)-HRESIMS [M + H]^+^ *m/z* 993.8342 (calcd for C_61_H_109_N_4_O_6_, 993.8342).

#### 3.1.13. *N*^1^,*N*^8^-Bis(3-((*R*)-4-((3*R*,5*S*,7*S*,8*R*,9*S*,10*S*,13*R*,14*S*,17*R*)-3,7-dihydroxy-10,13-dimethylhexadecahydro-1*H*-cyclopenta[*a*]phenanthren-17-yl)pentanamido)propyl)octane-1,8-diaminium 2,2,2-trifluoroacetate (**8d**)

Following general procedure B, ursodeoxycholic acid (0.050 g, 0.127 mmol) was reacted with di-*tert*-butyl octane-1,8-diylbis((3-aminopropyl)carbamate) (**6d**) (0.029 g, 0.0637 mmol), EDC·HCl (0.037 g, 0.191 mmol), HOBt (0.009 g, 0.0637 mmol) and DIPEA (0.044 mL, 0.255 mmol) to afford di-*tert*-butyl octane-1,8-diylbis((3-((*R*)-4-((3*R*,5*S*,7*S*,8*R*,9*S*,10*S*,13*R*,14*S*,17*R*)-3,7-dihydroxy-10,13-dimethylhexadecahydro-1*H*-cyclopenta[*a*]phenanthren-17-yl)pentanamido)propyl)carbamate) (0.067 g, 87%) as a white foam. Following general procedure C, a sub-sample of the product (0.026 g, 0.0215 mmol) was reacted with TFA in CH_2_Cl_2_ to afford, after chromatography, the di-TFA salt **8d** (0.025 g, 94%) as a pale-yellow oil. [α${]}_{D}^{20.4}$ = +20 (*c* = 0.1, MeOH); R*_f_* (RP-18, 10% *aq*. HCl:MeOH 1:3) 0.23; IR (ATR) ν_max_ 3310, 2933, 2865, 1635, 1454, 1202, 1137, 1015 cm^−1^; ^1^H NMR (DMSO-*d*_6_, 400 MHz) δ 8.48 (4H, br s, NH_2_-29), 7.89 (2H, t, *J* = 5.7 Hz, NH-25), 3.35–3.25 (4H, m, H-3, H-7), 3.10 (4H, q, *J* = 6.3 Hz, H_2_-26), 2.86 (8H, br s, H_2_-28, H_2_-30), 2.15–2.07 (2H, m, H_2_-23b), 2.02–1.95 (2H, m, H_2_-23a), 1.92–0.91 (64H, m, H_2_-1, H_2_-2, H_2_-4, H-5, H_2_-6, H-8, H-9, H_2_-11, H_2_-12, H-14, H_2_-15, H_2_-16, H-17, H-20, H_2_-22, H_2_-27, H_2_-31, H_2_-32, H_2_-33), 0.88 (6H, d, *J* = 6.5 Hz, H_3_-21), 0.87 (6H, s, H_3_-19), 0.61 (6H, s, H_3_-18); ^13^C NMR (DMSO-*d*_6_, 100 MHz) δ 173.3 (C-24), 69.7 (C-3), 69.5 (C-7), 55.9 (C-14), 54.7 (C-17), 46.8 (C-30), 44.6 (C-28), 43.1, 43.0 (C-8, C-13), 42.1 (C-5), 39.4 (C-12, obscured by solvent), 38.7 (C-10), 37.7 (C-4), 37.2 (C-6), 35.5 (C-26), 35.0, 34.8 (C-9, C-15, C-20), 33.7 (C-1), 32.3 (C-23), 31.6 (C-22), 30.2 (C-2), 28.3 (C-33), 28.2 (C-16), 26.2 (C-27), 25.8 (C-32), 25.4 (C-31), 23.3 (C-19), 20.8 (C-11), 18.4 (C-21), 12.0 (C-18); (+)-HRESIMS [M + 2H]^2+^ *m/z* 504.4260 (calcd for C_62_H_112_N_4_O_6_, 504.4285).

#### 3.1.14. *N*^1^,*N*^10^-Bis(3-((*R*)-4-((3*R*,5*S*,7*S*,8*R*,9*S*,10*S*,13*R*,14*S*,17*R*)-3,7-dihydroxy-10,13-dimethylhexadecahydro-1*H*-cyclopenta[*a*]phenanthren-17-yl)pentanamido)propyl)decane-1,10-diaminium 2,2,2-trifluoroacetate (**8e**)

Following general procedure B, ursodeoxycholic acid (0.034 g, 0.0886 mmol) was reacted with di-*tert*-butyl decane-1,10-diylbis((3-aminopropyl)carbamate) (**6e**) (0.021 g, 0.0443 mmol), EDC·HCl (0.025 g, 0.130 mmol), HOBt (0.006 g, 0.0443 mmol) and DIPEA (0.030 mL, 0.173 mmol) to afford di-*tert*-butyl decane-1,10-diylbis((3-((*R*)-4-((3*R*,5*S*,7*S*,8*R*,9*S*,10*S*,13*R*,14*S*,17*R*)-3,7-dihydroxy-10,13-dimethylhexadecahydro-1*H*-cyclopenta[*a*] phenanthren-17-yl)pentanamido)propyl) carbamate) as a clear colorless oil (0.044 g, 82%). Following general procedure C, a sub-sample of the product (0.009 g, 0.00728 mmol) was reacted with TFA in CH_2_Cl_2_ to afford, after chromatography, the di-TFA salt **8e** (0.006 g, 65%) as a pale-yellow oil. [α${]}_{D}^{18.3}$ = +33 (*c* = 0.1, MeOH); R*_f_* (RP-18, 10% *aq*. HCl:MeOH 1:3) 0.19; IR (ATR) ν_max_ 3363, 2931, 2861, 1676, 1452, 1202, 1136, 1016, 953, 801, 722 cm^−1^; ^1^H NMR (DMSO-*d*_6_, 400 MHz) δ 8.38 (4H, br s, NH_2_-29), 7.98 (2H, t, *J* = 5.7 Hz, NH-25), 3.36–3.23 (4H, m, H-3, H-7), 3.10 (4H, q, *J* = 6.3 Hz, H_2_-26), 2.85 (8H, br s, H_2_-28, H_2_-30), 2.15–2.08 (2H, m, H_2_-23b), 2.02–1.96 (2H, m, H_2_-23a), 1.94–0.91 (68H, m, H_2_-1, H_2_-2, H_2_-4, H-5, H_2_-6, H-8, H-9, H_2_-11, H_2_-12, H-14, H_2_-15, H_2_-16, H-17, H-20, H_2_-22, H_2_-27, H_2_-31, H_2_-32, H_2_-33, H_2_-34), 0.89 (6H, d, *J* = 6.6 Hz, H_3_-21), 0.87 (6H, s, H_3_-19), 0.61 (6H, s, H_3_-18); ^13^C NMR (DMSO-*d*_6_, 100 MHz) δ 173.3 (C-24), 69.7 (C-3), 69.4 (C-7), 55.9 (C-14), 54.7 (C-17), 46.8 (C-30), 44.5 (C-28), 43.1, 43.0 (C-8, C-13), 42.1 (C-5), 39.4 (C-12, obscured by solvent), 38.7 (C-10), 37.7 (C-4), 37.2 (C-6), 35.4 (C-26), 35.0, 34.8 (C-9, C-15, C-20), 33.7 (C-1), 32.3 (C-23), 31.6 (C-22), 30.2 (C-2), 28.8 (C-34), 28.5 (C-33), 28.2 (C-16), 26.2 (C-27), 25.9 (C-32), 25.5 (C-31), 23.3 (C-19), 20.8 (C-11), 18.4 (C-21), 12.0 (C-18); (+)-HRESIMS [M + 2H]^2+^ *m/z* 518.4441 (calcd for C_64_H_116_N_4_O_6_, 518.4442).

#### 3.1.15. *N*^1^,*N*^12^-Bis(3-((*R*)-4-((3*R*,5*S*,7*S*,8*R*,9*S*,10*S*,13*R*,14*S*,17*R*)-3,7-dihydroxy-10,13-dimethylhexadecahydro-1*H*-cyclopenta[*a*]phenanthren-17-yl)pentanamido)propyl)dodecane-1,12-diaminium 2,2,2-trifluoroacetate (**8f**)

Following general procedure B, ursodeoxycholic acid (0.050 g, 0.127 mmol) was reacted with di-*tert*-butyl dodecane-1,12-diylbis((3-aminopropyl)carbamate) (**6f**) (0.033 g, 0.0637 mmol), EDC·HCl (0.037 g, 0.191 mmol), HOBt (0.009 g, 0.0637 mmol) and DIPEA (0.044 mL, 0.255 mmol) to afford di-*tert*-butyl dodecane-1,12-diylbis((3-((*R*)-4-((3*R*,5*S*,7*S*,8*R*,9*S*,10*S*,13*R*,14*S*,17*R*)-3,7-dihydroxy-10,13-dimethylhexadecahydro-1*H*-cyclopenta[*a*]phenanthren-17-yl)pentanamido)propyl)carbamate) (0.066 g, 82%) as a white foam. Following general procedure C, a sub-sample of the product (0.009 g, 0.00712 mmol) was reacted with TFA in CH_2_Cl_2_ to afford, after chromatography, the di-TFA salt **8f** (0.007 g, 76%) as a pale-yellow oil. ${[\alpha ]}_{D}^{18.6}$ = +27 (*c* = 0.1, MeOH); R*_f_* (RP-18, 10% *aq*. HCl:MeOH 1:3) 0.17; IR (ATR) ν_max_ 3309, 2930, 2852, 1636, 1449, 1202, 1038, 1026 cm^−1^; ^1^H NMR (DMSO-*d*_6_, 400 MHz) δ 9.00 (4H, br s, NH_2_-29), 8.11 (2H, br s, NH-25), 3.34–3.23 (4H, m, H-3, H-7), 3.08 (4H, q, *J* = 6.2 Hz, H_2_-26), 2.80 (8H, br s, H_2_-28, H_2_-30), 2.14–2.07 (2H, m, H_2_-23b), 2.02–1.96 (2H, m, H_2_-23a), 1.93–0.93 (72H, m, H_2_-1, H_2_-2, H_2_-4, H-5, H_2_-6, H-8, H-9, H_2_-11, H_2_-12, H-14, H_2_-15, H_2_-16, H-17, H-20, H_2_-22, H_2_-27, H_2_-31, H_2_-32, H_2_-33, H_2_-34, H_2_-35), 0.87 (6H, d, *J* = 6.5 Hz, H_3_-21), 0.85 (6H, s, H_3_-19), 0.59 (6H, s, H_3_-18); ^13^C NMR (DMSO-*d*_6_, 100 MHz) δ 173.3 (C-24), 69.8 (C-3), 69.5 (C-7), 55.9 (C-14), 54.8 (C-17), 46.8 (C-30), 44.5 (C-28), 43.2, 43.1 (C-8, C-13), 42.2 (C-5), 39.8 (C-10, obscured by solvent), 39.2 (C-12, obscured by solvent), 38.8 (C-10), 37.8 (C-4), 37.3 (C-6), 35.7 (C-26), 35.1, 34.9 (C-9, C-15, C-20), 33.8 (C-1), 32.4 (C-23), 31.7 (C-22), 30.3 (C-2), 29.0 (C-35), 28.9 (C-34), 28.6 (C-33), 28.3 (C-16), 26.1 (C-27), 25.9 (C-32), 25.4 (C-31), 23.4 (C-19), 20.9 (C-11), 18.5 (C-21), 12.1 (C-18); (+)-HRESIMS [M + H]^+^ *m/z* 1063.9124 (calcd for C_66_H_119_N_4_O_6_, 1063.9124).

#### 3.1.16. *N*^1^,*N*^4^-Bis(3-((*R*)-4-((3*R*,5*S*,7*R*,8*R*,9*S*,10*S*,13*R*,14*S*,17*R*)-3,7-dihydroxy-10,13-dimethylhexadecahydro-1*H*-cyclopenta[*a*]phenanthren-17-yl)pentanamido)propyl)butane-1,4-diaminium 2,2,2-trifluoroacetate (**9a**)

Following general procedure B, chenodeoxycholic acid (0.050 g, 0.127 mmol) was reacted with di-*tert*-butyl butane-1,4-diylbis((3-aminopropyl)carbamate) (**6a**) (0.026 g, 0.0637 mmol), EDC·HCl (0.037 g, 0.191 mmol), HOBt (0.009 g, 0.0637 mmol) and DIPEA (0.044 mL, 0.255 mmol) to afford di-*tert*-butyl butane-1,4-diylbis((3-((*R*)-4-((3*R*,5*S*,7*R*,8*R*,9*S*,10*S*,13*R*,14*S*,17*R*)-3,7-dihydroxy-10,13-dimethylhexadecahydro-1*H*-cyclopenta[*a*]phenanthren-17-yl)pentanamido)propyl)carbamate) (0.060 g, 82%) as a clear colorless oil. Following general procedure C, a sub-sample of the product (0.030 g, 0.0260 mmol) was reacted with TFA in CH_2_Cl_2_ to afford, after chromatography, the di-TFA salt **9a** (0.018 g, 59%) as a clear, colorless oil. [α]_D_ = +8 (*c* = 0.1, MeOH); R*_f_* (RP-18, 10% *aq*. HCl:MeOH 1:3) 0.11; IR (ATR) ν_max_ 3308, 2961, 2838, 1645, 1410, 1259, 1092, 1038, 1015, 798 cm^−1^; ^1^H NMR (DMSO-*d*_6_, 400 MHz) δ 8.46 (4H, br s, NH_2_-29), 7.98 (2H, t, *J* = 5.7 Hz, NH-25), 4.01 (2H, br s, OH-7), 3.62 (2H, br s, H-7), 3.22–3.15 (2H, m, H-3), 3.10 (8H, q, *J* = 6.3 Hz, H_2_-28, H_2_-30), 2.92–2.83 (4H, m, H_2_-26), 2.23–2.14 (2H, m, H_2_-4a), 2.12–2.08 (2H, m, H_2_-23b), 2.02–1.96 (2H, m, H_2_-23a), 1.95–0.96 (54H, m, H_2_-1, H_2_-2, H_2_-4b, H-5, H_2_-6, H-8, H-9, H_2_-11, H_2_-12, H-14, H_2_-15, H_2_-16, H-17, H-20, H_2_-22, H_2_-27, H_2_-31), 0.89 (6H, d, *J* = 6.4 Hz, H_3_-21), 0.83 (6H, s, H_3_-19), 0.60 (6H, s, H_3_-18); ^13^C NMR (DMSO-*d*_6_, 100 MHz) δ 173.3 (C-24), 70.3 (C-3), 66.1 (C-7), 55.5 (C-17), 50.0 (C-14), 46.1 (C-30), 44.6 (C-28), 41.9 (C-8), 41.4 (C-5), 40.5 (C-13), 39.8 (C-4, obscured by solvent), 39.2 (C-9 and C-12, obscured by solvent), 35.5 (C-26), 35.3 (C-6), 35.1, 34.8 (C-10, C-16), 34.7 (C-20), 32.3 (C-1, C-23), 31.5 (C-22), 30.5 (C-2), 26.2 (C-27), 23.1 (C-15), 22.7 (C-19, C-31), 20.2 (C-11), 18.3 (C-21), 11.6 (C-18); (+)-HRESIMS [M + H]^+^ *m/z* 951.7874 (calcd for C_58_H_103_N_4_O_6_, 951.7872).

### 3.2. Antimicrobial Assays

Antimicrobial evaluation against *S. aureus* (MRSA) (ATCC 43300), *E. coli* (ATCC 25922), *K. pneumoniae* (ATCC 700603), *A. baumannii* (ATCC 19606), *C. albicans* (ATCC 90028), and *C. neoformans* (ATCC 208821) was undertaken at the Community for Open Antimicrobial Drug Discovery at The University of Queensland (Australia) according to their standard protocols as reported previously [32,33].

Additional antimicrobial evaluation against *S. aureus* (ATCC 25923), *S. aureus* MRSA (CF-Marseille) and Bacillus cereus (ATCC 11778) was determined in microplates using the standard broth dilution method in accordance with the recommendations of the Comité de l’AntibioGramme de la Société Française de Microbiologie (CA-SFM). Briefly, the minimal inhibitory concentrations (MICs) were determined with an inoculum of 10^5^ CFU in 200 µL of MHB containing two-fold serial dilutions of each drug. The MIC was defined as the lowest concentration of drug that completely inhibited visible growth after incubation for 18 h at 37 °C. To determine all MICs, the measurements were independently repeated in triplicate.

### 3.3. Cytotoxicity Assays

Cytotoxicity assays were conducted using the protocols previously reported [33].

### 3.4. Hemolytic Assays

Hemolytic assays were conducted using the protocols previously reported [33].

### 3.5. Real Time Growth Curves

Solutions of compound **7e** at concentrations of 2, 4 and 16 µg/mL were tested each in triplicate against *S. aureus* ATCC 25923, *S. aureus* MRSA (CF-Marseille) and *Bacillus cereus* ATCC 11778. Typically, in a 96 well plate was placed 10 µL of 40, 80 and 320 µg/mL stock solutions of compound **7e** as well as 190 µL of a 5 × 10^5^ CFU/mL of the selected bacterial suspension in brain heart infusion (BHI) broth. Positive controls containing only 200 µL of a 5 × 10^5^ CFU/mL of bacterial suspension in BHI and negative controls containing only 200 µL of BHI broth were added. The plate was incubated at 37 °C in a TECAN Spark Reader (Roche Diagnostic) and real time bacterial growth was followed with OD_590_ nm measurements every 10 min during 19 h.

### 3.6. Minimum Bactericidal Concentration Test

A pure culture of a specified microorganism was grown overnight, then diluted in growth-supporting broth (typically Mueller Hinton II broth) to a concentration between 1 × 10^5^ and 1 × 10^6^ CFU/mL. A stock dilution of the antimicrobial test compound was made at approximately 100 times the expected, previously determined MIC. Further 1:1 dilution was made in 96 well microtiter plates. All dilutions of the test compound were inoculated with equal volumes of the specified microorganism (typically 100 µL). A positive and negative control tube or well was included to demonstrate adequate microbial growth over the course of the incubation period and media sterility, respectively. An aliquot of the positive control was plated and used to establish a baseline concentration of the microorganism used. The microtiter plates were then incubated at 37 °C for 24 h. Turbidity indicates growth of the microorganism, and the MIC is the lowest concentration where no growth was visually observed. To determine the minimum bactericidal concentration (MBC), the dilution representing the MIC and at least two of the more concentrated test product dilutions was plated on a solidified agar plate to determine the bacterial viability. The MBC is the lowest concentration where no growth is encountered when compared to the MIC dilution.

### 3.7. ATP Release Assay

Solutions of the test compound **7e** were prepared in DMSO at various concentrations. A suspension of growing *S. aureus* to be studied in Muller-Hinton II broth was prepared and incubated at 37 °C. An aliquot (90 µL) of this suspension was added to 10 µL of test compound solution and vortexed for 10 s. Luciferin–luciferase reagent (Yelen, France; 50 µL) was immediately added to this mixture, and luminescent signal quantified with an Infinite M200 microplate reader (Tecan) over a 30 min period; ATP concentration was quantified using internal sample addition. A similar procedure was used for the CTAB positive control.

### 3.8. Determination of Antibiotic Enhancement

Restoring enhancer concentrations were determined with an inoculum of 5 × 10^5^ CFU in 200 mL of MH broth containing two-fold serial dilutions of each derivative in the presence of either doxycycline or erythromycin at 2 μg/mL. The lowest concentration of the polyamine adjuvant that completely inhibited visible growth after incubation for 18 h at 37 °C was determined. These measurements were independently repeated in triplicate.

## 4. Conclusions

In summary, we have synthesized a focused set of dimeric deoxycholic acid-based polyamine derivatives that explored variation in the cholic acid head group (hyodeoxycholic acid, ursodeoxycholic acid and chenodeoxycholic acid) as well as variation in polyamine chain length. Preliminary antimicrobial activities were evaluated against one Gram-positive (*S. aureus* MRSA), four Gram-negative (*E. coli*, *P. aeruginosa*, *A. baumannii*, *K. pneumoniae*) bacteria and two fungi (*Candida albicans* and *Cryptococcus neoformans*). Many of the compounds exhibited pronounced activity towards *S. aureus* MRSA with some also exhibiting potent antifungal activity. The overall set of analogues were noticeably inactive against all the target Gram-negative bacteria. Counter-screening for toxicity indicators identified HDCA analogues **7a**–**f** and the sole CDCA analogue **9a** to be devoid of cytotoxicity towards the HEK293 and to be non-hemolytic. The observation of cytotoxicity and/or hemolytic activities for the UDCA analogues **8a**–**f** indicates quite a precise structural requirement for bacterial *versus* mammalian cell toxicity. HDCA analogue **7e** exhibited an MBC/MIC ratio of approximately one against three Gram-positive bacteria strains, identifying it to be a bactericidal agent. In a preliminary evaluation of its mechanism of action, **7e** failed to cause ATP release from *S. aureus* cells, a somewhat surprising result given squalamine and many squalamine mimics are reported to target and disrupt bacterial membranes. Together, the present study identifies HDCA-polyamine analogues as being worthy of further study as potent, non-toxic, Gram-positive bactericides with a seemingly unexpected mechanism of action.

## Figures and Tables

**Table 1:** Antimicrobial (MIC, μM) activities^1^ of compounds **7**–**9** and squalamine (**1**).

Compound	S.a. ^a^	E.c. ^b^	K.p. ^c^	P.a. ^d^	A.b. ^e^	C.a. ^f^	C.n. ^g^
**7a**	3.4	>27	>27	>27	>27	14	≤0.21
**7b**	≤0.21	>26	>26	>26	>26	13	≤0.21
**7c**	≤0.21	>26	>26	>26	>26	13	≤0.21
**7d**	≤0.20	>26	>26	>26	>26	13	≤0.20
**7e**	≤0.20	>25	>25	>25	>25	≤0.20	0.80
**7f**	≤0.19	>25	>25	>25	>25	≤0.19	≤0.19
**8a**	27	>27	>27	>27	>27	27	>27
**8b**	1.7	>26	>26	>26	>26	27	>27
**8c**	≤0.21	>26	>26	>26	>26	13	26
**8d**	≤0.20	>26	>26	>26	>26	26	13
**8e**	≤0.20	>25	>25	>25	>25	3.2	1.6
**8f**	≤0.19	>25	>25	>25	>25	3.1	≤0.19
**9a**	≤0.21	6.8	>27	>27	27	≤0.21	≤0.21
**1**	≤0.28	≤0.28	≤0.28	18	≤0.28	4.5	0.56

All values presented as the mean (n = 2). ^a^ *Staphylococcus aureus* ATCC 43300 (MRSA) with vancomycin (MIC 0.7 μM) used as a positive control; ^b^ Escherichia coli ATCC 25922 with colistin (MIC0.1 μM); ^c^ Klebsiella pneumoniae ATCC 700603 with colistin (MIC 0.2 μM); ^d^ *Pseudomonas aeruginosa* ATCC 27853 with colistin (MIC 0.2 μM); ^e^ *Acinetobacter baumannii* ATCC 19606 with colistin (MIC0.2 μM); ^f^ *Candida albicans* ATCC 90028 with fluconazole (MIC 0.4 μM) as a positive control; ^g^ *Cryptococcus neoformans* ATCC 208821 with fluconazole (MIC 26 μM) as a positive control.

**Table 2:** Cytotoxicity (IC_50_, μM) and hemolytic (HC_10_, μM) activities of compounds **7**–**9** and squalamine (**1**).

Compound	Cytotoxicity ^a^	Hemolysis ^b^
**7a**	>27	>27
**7b**	>26	>26
**7c**	>26	>26
**7d**	>26	>26
**7e**	>25	>25
**7f**	25	>25
**8a**	>27	7.3
**8b**	>27	11
**8c**	>26	11
**8d**	>26	5.6
**8e**	>25	3.3
**8f**	8.4	1.4
**9a**	27	27
**1**	>36	>36

^a^ Concentration (μM) of compound at 50% cytotoxicity on HEK293 human embryonic kidney cells and values presented as the mean (n = 2). Tamoxifen was the positive control (IC_50_ 24 μM); ^b^ Concentration (μM) of compound at 10% hemolytic activity on human red blood cells and values presented as the mean (n = 2). Melittin was the positive control (HC_10_ 0.95 μM).

## Data Availability

Data are contained within the article or Supplementary Materials.

## Supplementary Materials

The following supporting information can be downloaded at: https://www.mdpi.com/article/10.3390/antibiotics12020404/s1, Figure S1: ^1^H (DMSO-*d*_6_, 400 MHz) and ^13^C (DMSO-*d*_6_, 100 MHz) NMR spectra for **7a**; Figure S2: ^1^H (DMSO-*d*_6_, 400 MHz) and ^13^C (DMSO-*d*_6_, 100 MHz) NMR spectra for **7b**; Figure S3: ^1^H (DMSO-*d*_6_, 400 MHz) and ^13^C (DMSO-*d*_6_, 100 MHz) NMR spectra for **7c**; Figure S4: ^1^H (DMSO-*d*_6_, 400 MHz) and ^13^C (DMSO-*d*_6_, 100 MHz) NMR spectra for **7d**; Figure S5: ^1^H (DMSO-*d*_6_, 400 MHz) and ^13^C (DMSO-*d*_6_, 100 MHz) NMR spectra for **7e**; Figure S6: ^1^H (DMSO-*d*_6_, 400 MHz) and ^13^C (DMSO-*d*_6_, 100 MHz) NMR spectra for **7f**; Figure S7: ^1^H (DMSO-*d*_6_, 400 MHz) and ^13^C (DMSO-*d*_6_, 100 MHz) NMR spectra for **8a**; Figure S8: ^1^H (DMSO-*d*_6_, 400 MHz) and ^13^C (DMSO-*d*_6_, 100 MHz) NMR spectra for **8b**; Figure S9: ^1^H (DMSO-*d*_6_, 400 MHz) and ^13^C (DMSO-*d*_6_, 100 MHz) NMR spectra for **8c**; Figure S10: ^1^H (DMSO-*d*_6_, 400 MHz) and ^13^C (DMSO-*d*_6_, 100 MHz) NMR spectra for **8d**; Figure S11: ^1^H (DMSO-*d*_6_, 400 MHz) and ^13^C (DMSO-*d*_6_, 100 MHz) NMR spectra for **8e**; Figure S12: ^1^H (DMSO-*d*_6_, 400 MHz) and ^13^C (DMSO-*d*_6_, 100 MHz) NMR spectra for **8f**; Figure S13: ^1^H (DMSO-*d*_6_, 400 MHz) and ^13^C (DMSO-*d*_6_, 100 MHz) NMR spectra for **9a**.
