# Supplementary material for: Investigation of α,ω-Disubstituted Polyamine-Cholic Acid Conjugates Identifies Hyodeoxycholic and Chenodeoxycholic Scaffolds as Non-Toxic, Potent Antimicrobials
Source: Antibiotics (Basel). 2023 Feb 17;12(2):404. doi: 10.3390/antibiotics12020404 (PMC9951859; doi:10.3390/antibiotics12020404)
Supplement: Supplementary file 1 [file antibiotics-12-00404-s001.zip › antibiotics-2223366-supplementary.pdf]

Supporting Information

Investigation of α,ω-disubstituted polyamine-cholic acid conjugates identifies hyodeoxycholic and chenodeoxycholic scaffolds as non-toxic, potent antimicrobials

Kenneth Sue [1], Melissa M. Cadelis [1], Thomas Troia [2], Florent Rouvier [2], Marie-Lise Bourguet-Kondracki [3], Jean Michel Brunel [2] and Brent R. Copp [1,*]

[1] School of Chemical Sciences, The University of Auckland, Private Bag 92019, Auckland 1142, New Zealand; ksue827@aucklanduni.ac.nz (K.S.); m.cadelis@auckland.ac.nz (M.M.C.); b.copp@auckland.ac.nz (B.C)
[2] UMR MD1 "Membranes et Cibles Therapeutiques", U1261 INSERM, Faculté de Pharmacie, Aix-Marseille Universite, 27 bd Jean Moulin, 13385 Marseille, France; troia.thomas@gmail.com (T.T.); rouv.flo@hotmail.fr (F.R.); jean-michel.brunel@inserm.fr (J.M.B.)
[3] Laboratoire Molécules de Communication et Adaptation des Micro-organismes, UMR 7245 CNRS, Muséum National d'Histoire Naturelle, 57 rue Cuvier (C.P. 54), 75005 Paris, France; marie-lise.bourguet@mnhn.fr (M-L.B-K)
* Correspondence: b.copp@auckland.ac.nz

Contents

Figure S1 ^{1}H (DMSO-d_6, 400 MHz) and ^{13}C (DMSO-d_6, 100 MHz) NMR spectra for **7a**. S2
Figure S2 ^{1}H (DMSO-d_6, 400 MHz) and ^{13}C (DMSO-d_6, 100 MHz) NMR spectra for **7b**. S3
Figure S3 ^{1}H (DMSO-d_6, 400 MHz) and ^{13}C (DMSO-d_6, 100 MHz) NMR spectra for **7c**. S4
Figure S4 ^{1}H (DMSO-d_6, 400 MHz) and ^{13}C (DMSO-d_6, 100 MHz) NMR spectra for **7d**. S5
Figure S5 ^{1}H (DMSO-d_6, 400 MHz) and ^{13}C (DMSO-d_6, 100 MHz) NMR spectra for **7e**. S6
Figure S6 ^{1}H (DMSO-d_6, 400 MHz) and ^{13}C (DMSO-d_6, 100 MHz) NMR spectra for **7f**. S7
Figure S7 ^{1}H (DMSO-d_6, 400 MHz) and ^{13}C (DMSO-d_6, 100 MHz) NMR spectra for **8a**. S8
Figure S8 ^{1}H (DMSO-d_6, 400 MHz) and ^{13}C (DMSO-d_6, 100 MHz) NMR spectra for **8b**. S9
Figure S9 ^{1}H (DMSO-d_6, 400 MHz) and ^{13}C (DMSO-d_6, 100 MHz) NMR spectra for **8c**. S10
Figure S10 ^{1}H (DMSO-d_6, 400 MHz) and ^{13}C (DMSO-d_6, 100 MHz) NMR spectra for **8d**. S11
Figure S11 ^{1}H (DMSO-d_6, 400 MHz) and ^{13}C (DMSO-d_6, 100 MHz) NMR spectra for **8e**. S12
Figure S12 ^{1}H (DMSO-d_6, 400 MHz) and ^{13}C (DMSO-d_6, 100 MHz) NMR spectra for **8f**. S13
Figure S13 ^{1}H (DMSO-d_6, 400 MHz) and ^{13}C (DMSO-d_6, 100 MHz) NMR spectra for **9a**. S14

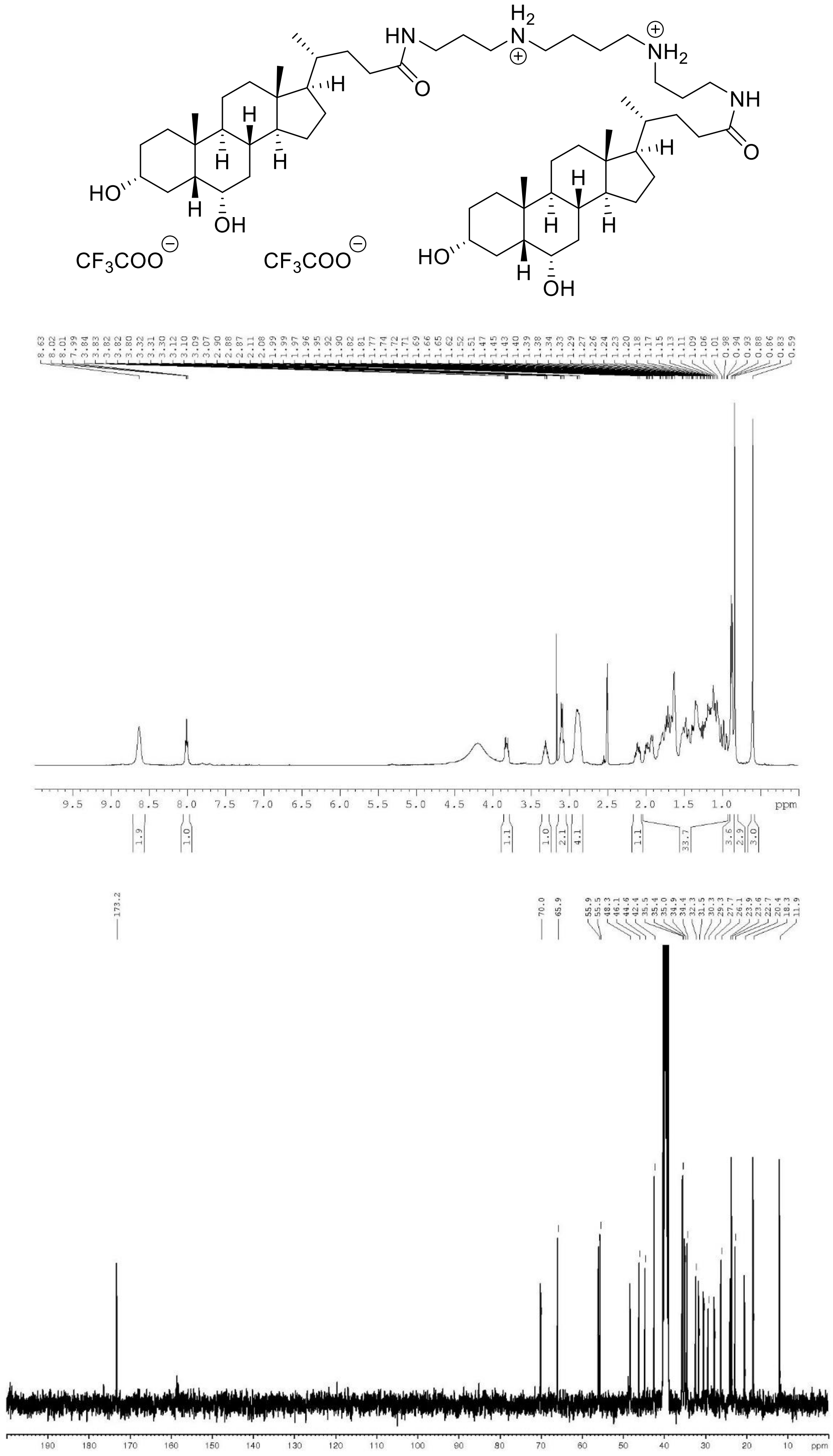

Figure S1 ^{1}H (DMSO-d_6, 400 MHz) and ^{13}C (DMSO-d_6, 100 MHz) NMR spectra for **7a**.

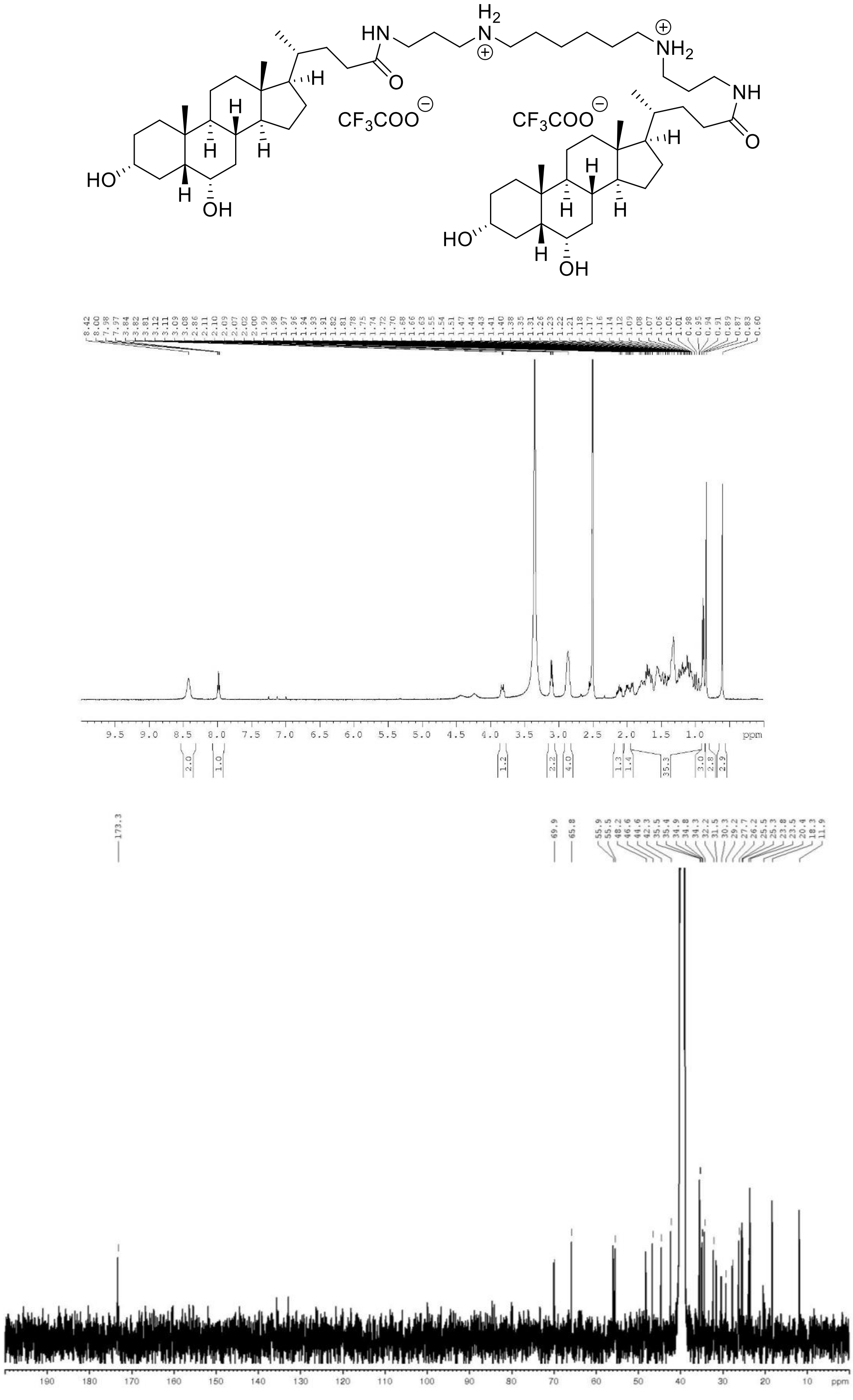

Figure S2 ^{1}H (DMSO-d_6, 400 MHz) and ^{13}C (DMSO-d_6, 100 MHz) NMR spectra for **7b**.

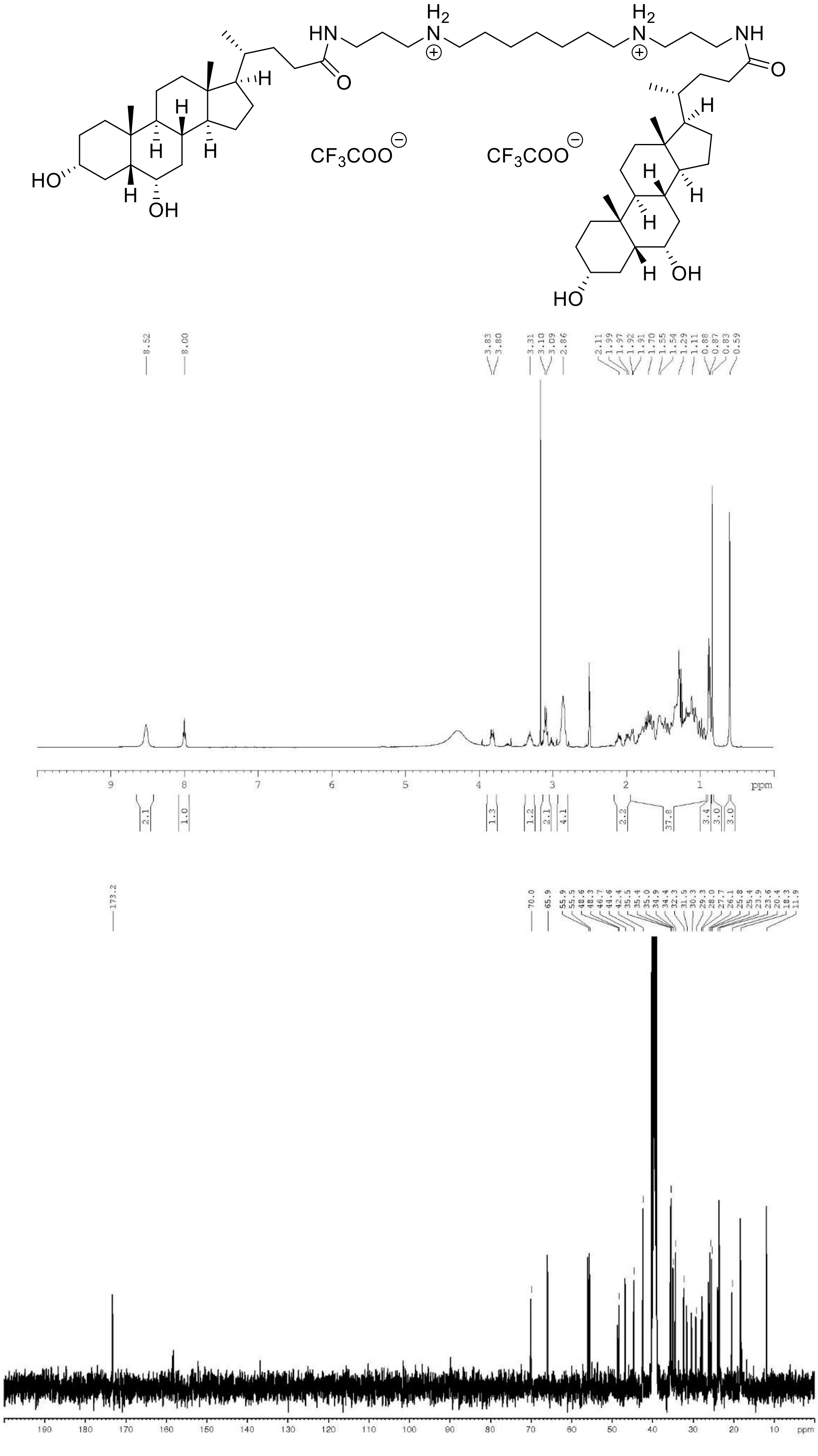

Figure S3 ^{1}H (DMSO-d_6, 400 MHz) and ^{13}C (DMSO-d_6, 100 MHz) NMR spectra for **7c**.

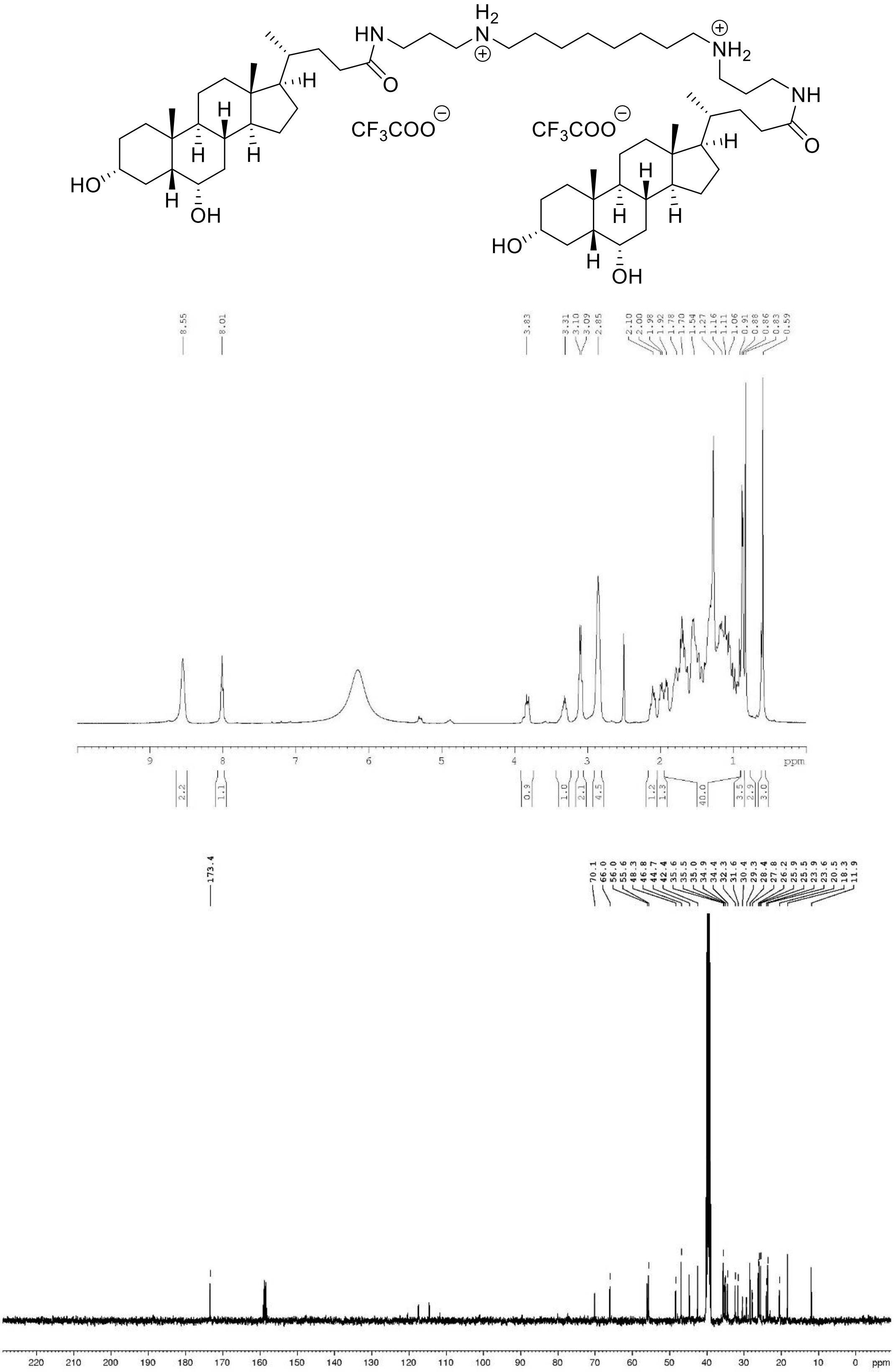

Figure S4 ^{1}H (DMSO-d_6, 400 MHz) and ^{13}C (DMSO-d_6, 100 MHz) NMR spectra for **7d**.

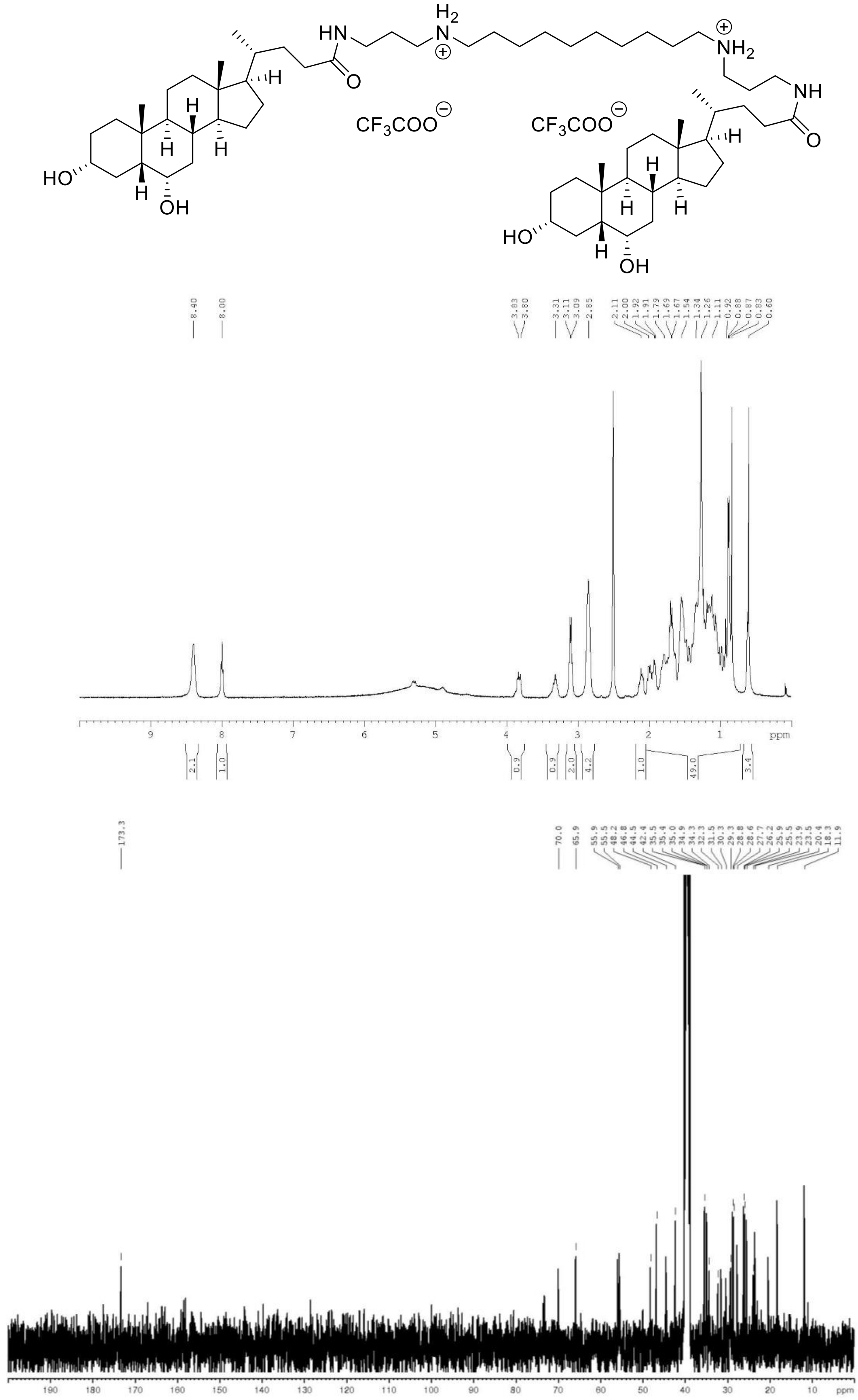

Figure S5 ^{1}H (DMSO-d_6, 400 MHz) and ^{13}C (DMSO-d_6, 100 MHz) NMR spectra for **7e**.

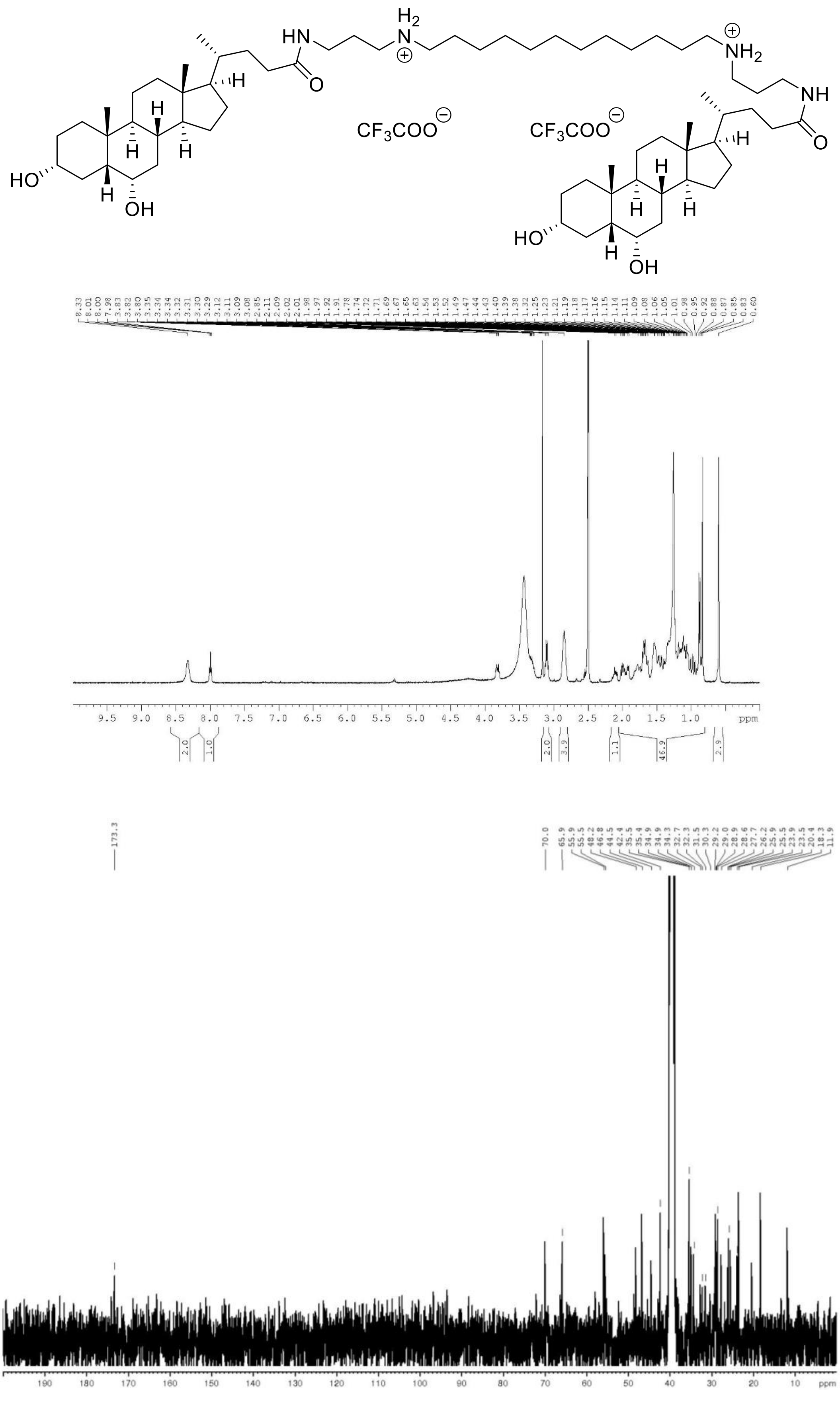

Figure S6 ^{1}H (DMSO-d_6, 400 MHz) and ^{13}C (DMSO-d_6, 100 MHz) NMR spectra for **7f**.

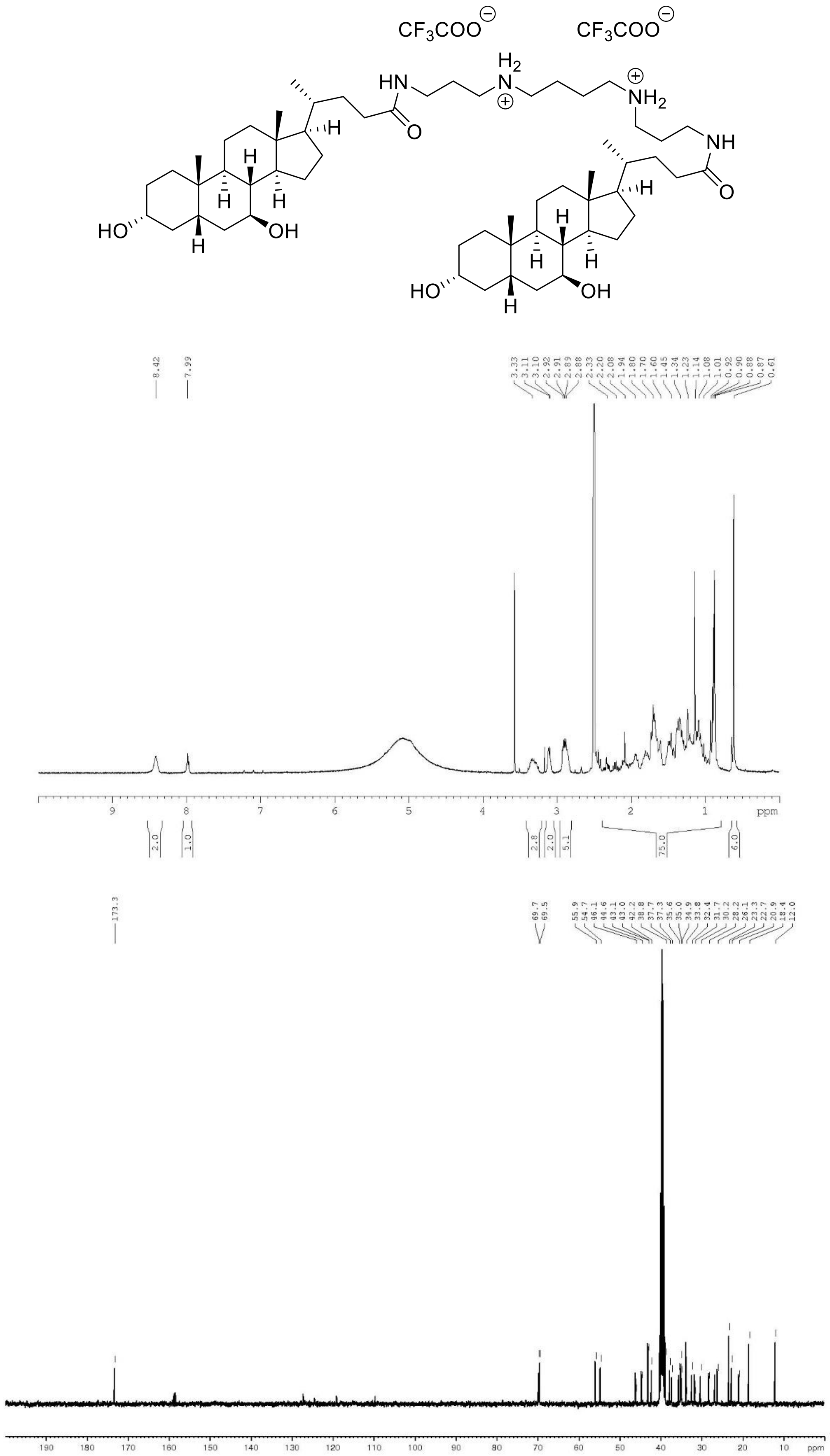

Figure S7 ^{1}H (DMSO-d_6, 400 MHz) and ^{13}C (DMSO-d_6, 100 MHz) NMR spectra for **8a**.

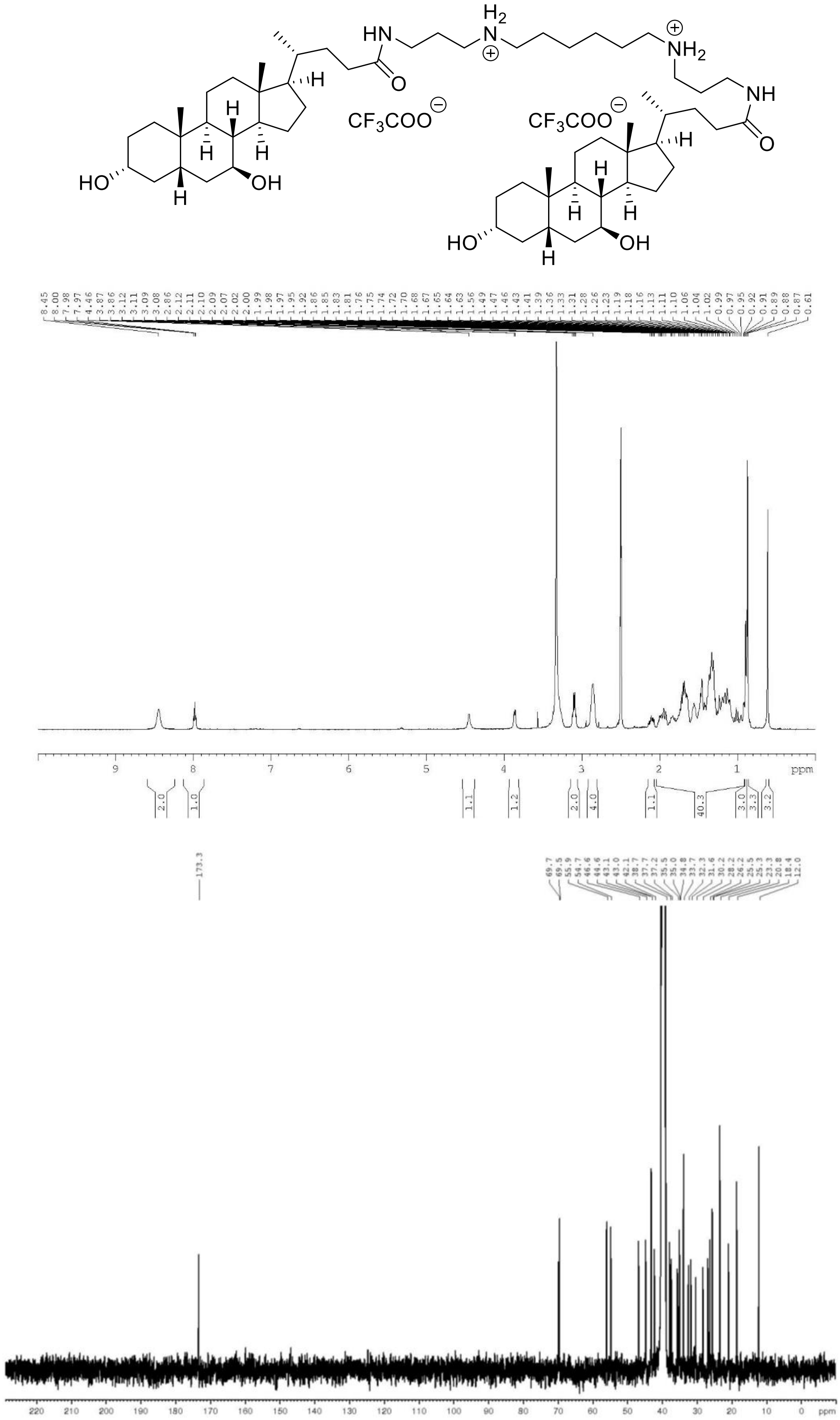

Figure S8 ^{1}H (DMSO-d_6, 400 MHz) and ^{13}C (DMSO-d_6, 100 MHz) NMR spectra for **8b**.

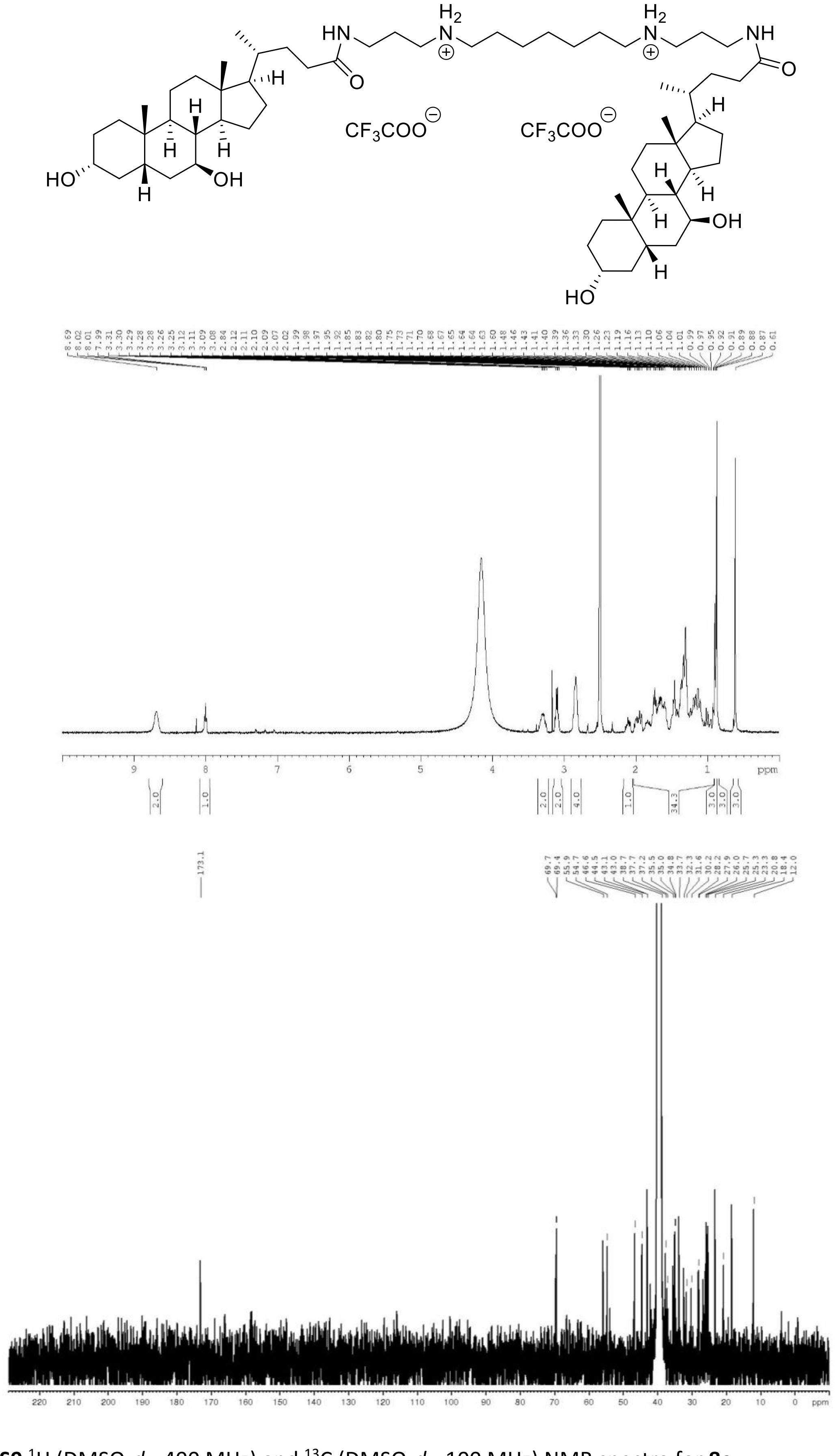

Figure S9 ^{1}H (DMSO-d_6, 400 MHz) and ^{13}C (DMSO-d_6, 100 MHz) NMR spectra for **8c**.

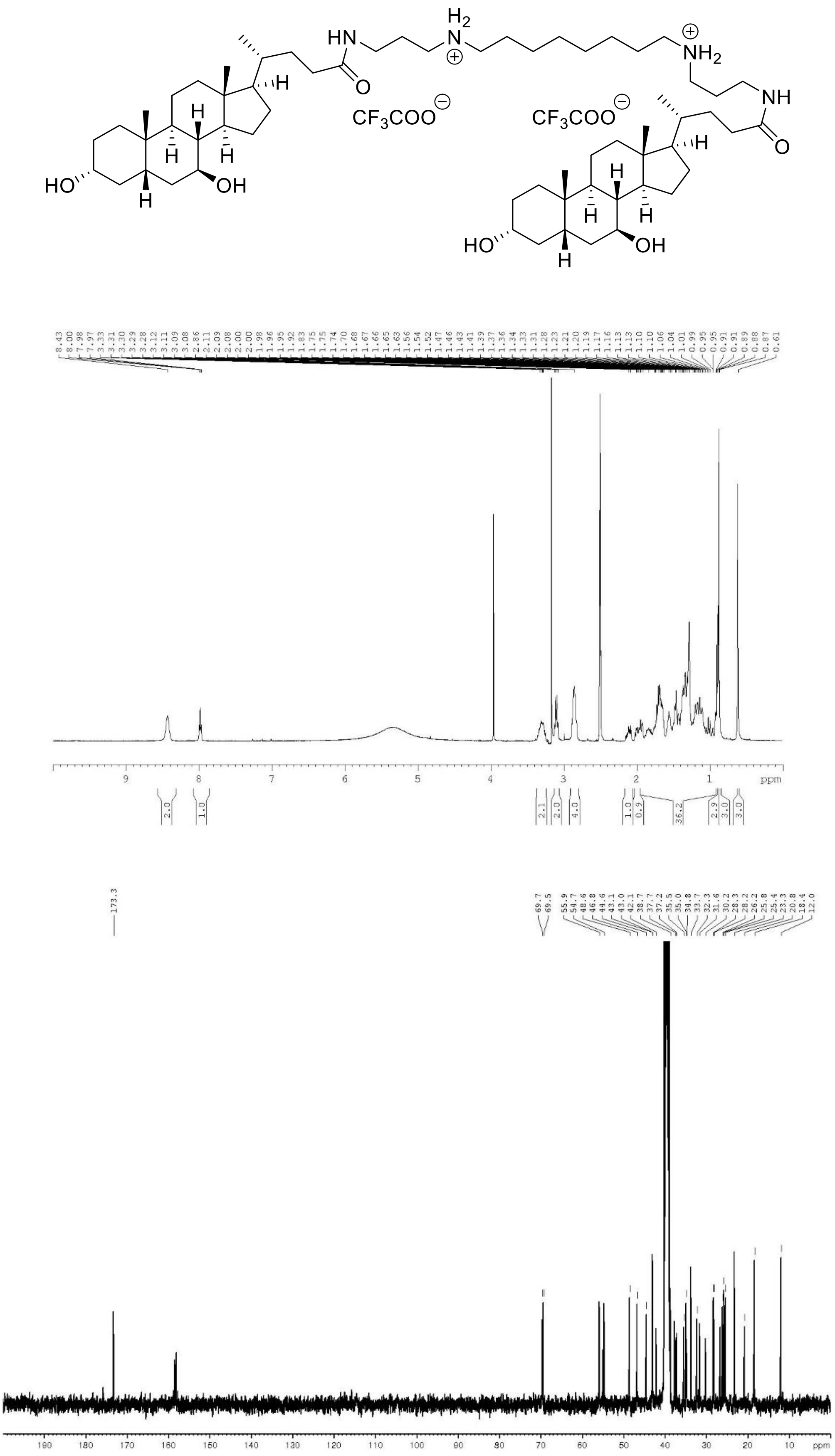

Figure S10 ^{1}H (DMSO-d_6, 400 MHz) and ^{13}C (DMSO-d_6, 100 MHz) NMR spectra for **8d**.

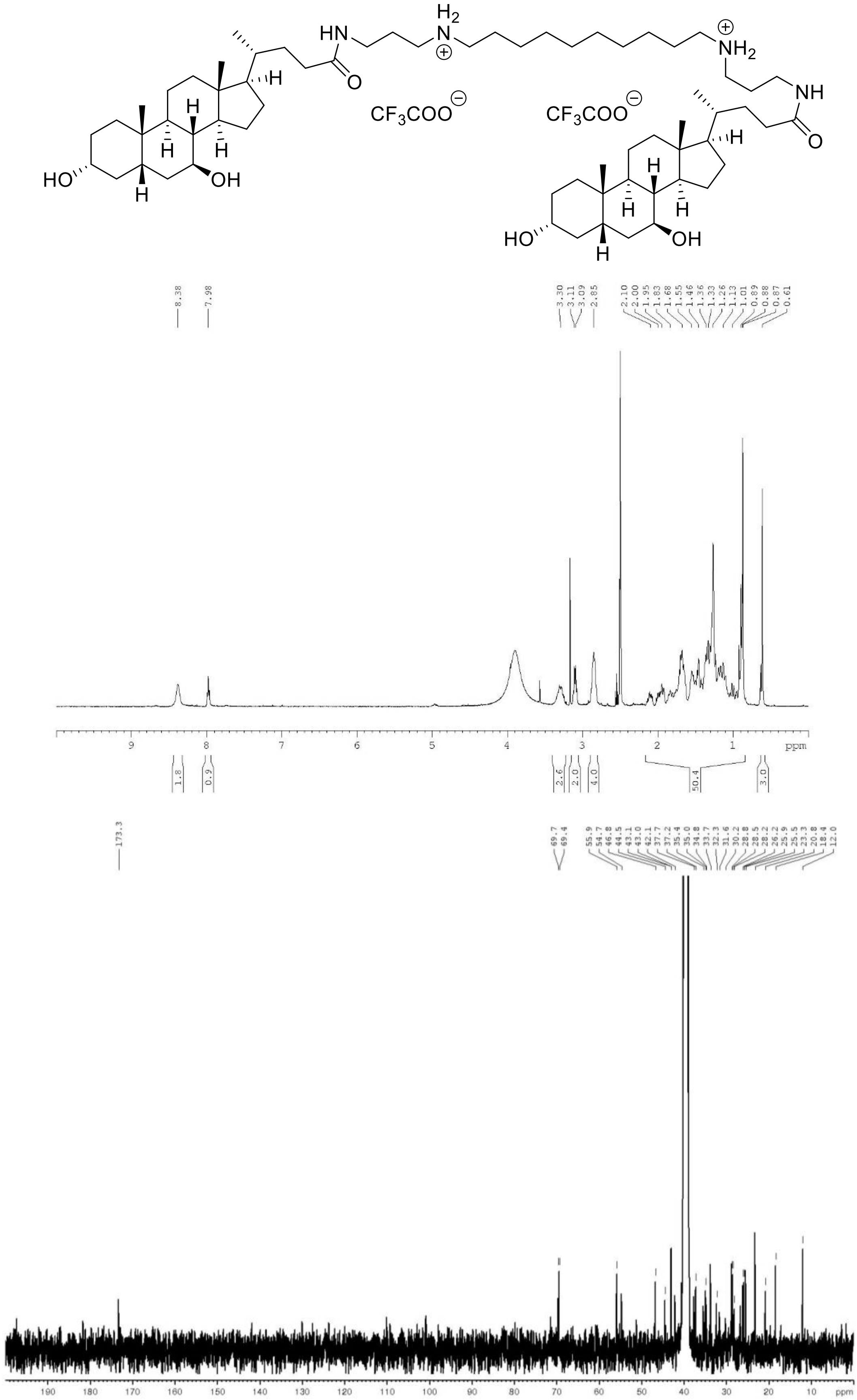

Figure S11 ^{1}H (DMSO-d_6, 400 MHz) and ^{13}C (DMSO-d_6, 100 MHz) NMR spectra for **8e**.

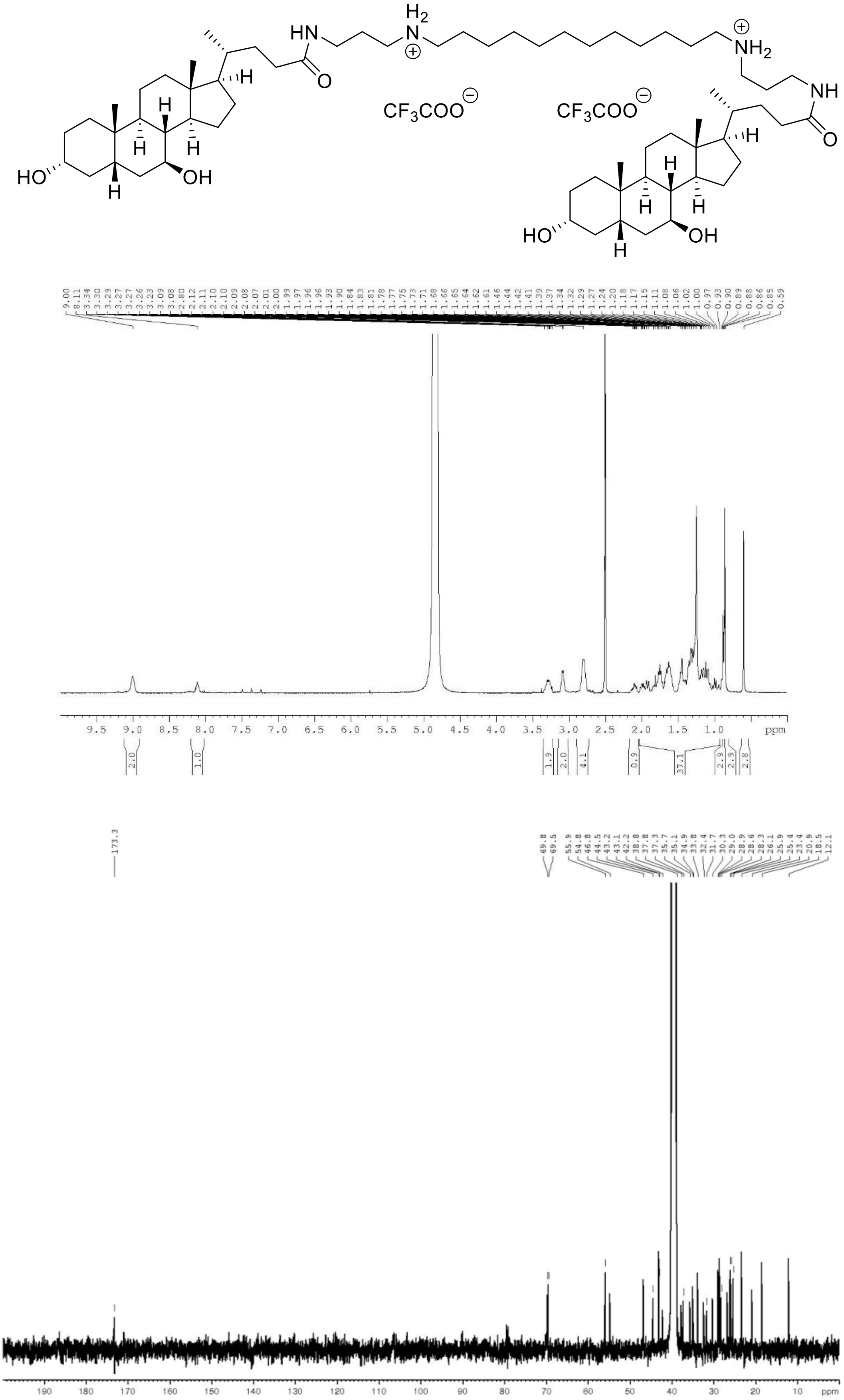

Figure S12 ^{1}H (DMSO-d_6, 400 MHz) and ^{13}C (DMSO-d_6, 100 MHz) NMR spectra for **8f**.

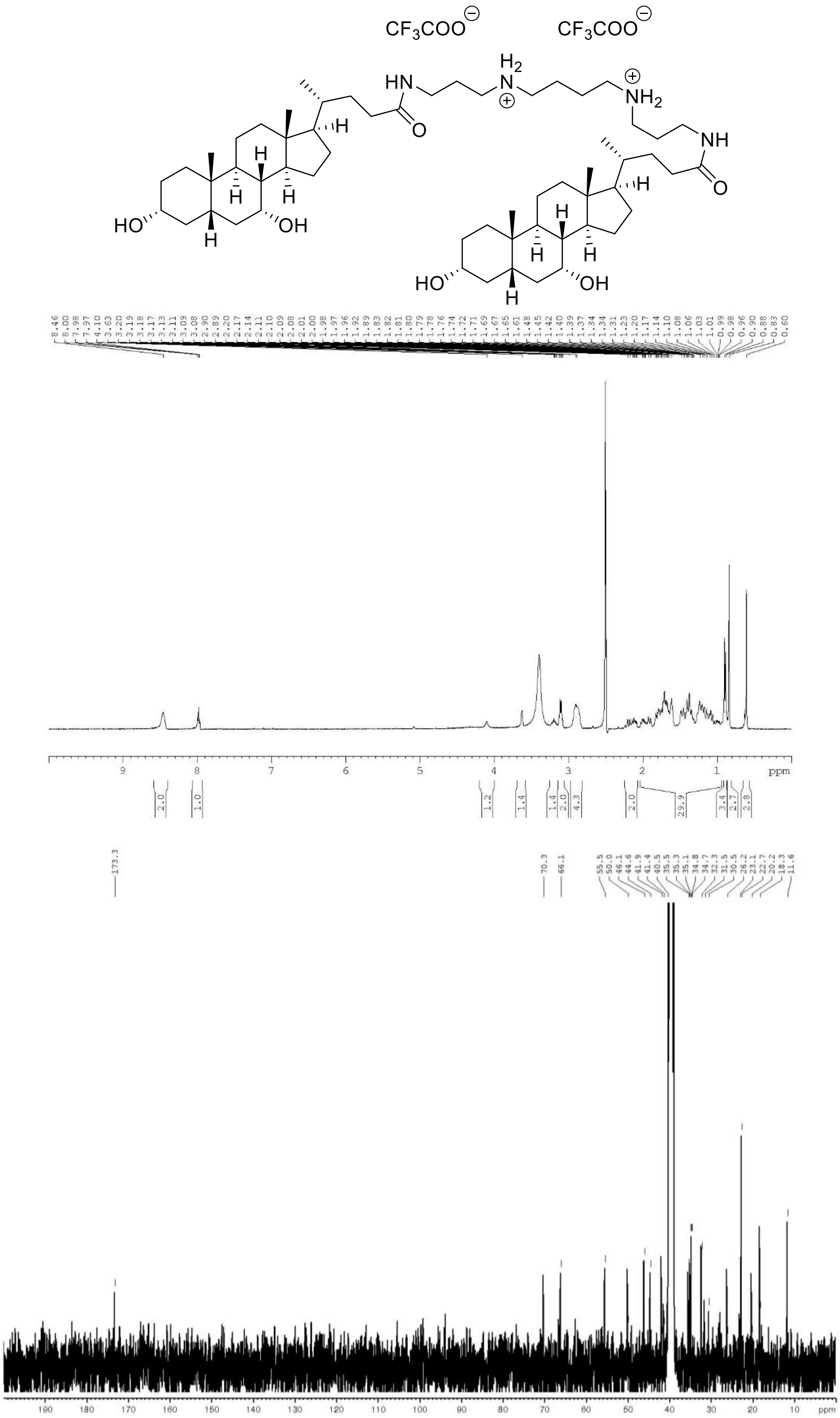

Figure S13 ^{1}H (DMSO-d_6, 400 MHz) and ^{13}C (DMSO-d_6, 100 MHz) NMR spectra for **9a**.
